# Remote light-controlled intracellular target recognition by photochromic fluorescent glycoprobes

**DOI:** 10.1038/s41467-017-01137-8

**Published:** 2017-10-17

**Authors:** Junji Zhang, Youxin Fu, Hai-Hao Han, Yi Zang, Jia Li, Xiao-Peng He, Ben L. Feringa, He Tian

**Affiliations:** 1 Key Laboratory for Advanced Materials & Institute of Fine Chemicals, School of Chemistry and Molecular Engineering, East China University of Science and Technology, 130 Meilong Rd., Shanghai, 200237 China; 2 National Center for Drug Screening, State Key Laboratory of Drug Research, Shanghai Institute of Materia Medica, Chinese Academy of Sciences, 189 Guo Shoujing Rd., Shanghai, 201203 China; 3 Centre for Systems Chemistry, Stratingh Institute for Chemistry and Zernike Institute for Advanced Materials, Faculty of Mathematics and Natural Sciences, University of Groningen, Nijenborgh 4, 9747 AG Groningen, The Netherlands

## Abstract

Development of powerful fluorescence imaging probes and techniques sets the basis for the spatiotemporal tracking of cells at different physiological and pathological stages. While current imaging approaches rely on passive probe–analyte interactions, here we develop photochromic fluorescent glycoprobes capable of remote light-controlled intracellular target recognition. Conjugation between a fluorophore and spiropyran produces the photochromic probe, which is subsequently equipped with a glycoligand “antenna” to actively localize a target cell expressing a selective receptor. We demonstrate that the amphiphilic glycoprobes that form micelles in water can selectively enter the target cell to operate photochromic cycling as controlled by alternate UV/Vis irradiations. We further show that remote light conversion of the photochromic probe from one isomeric state to the other activates its reactivity toward a target intracellular analyte, producing locked fluorescence that is no longer photoisomerizable. We envision that this research may spur the use of photochromism for the development of bioimaging probes.

## Abstract (web summary)

Fluorescence sensing in biological environments is prone to background signal interference. Here the authors design a photochromic fluorescent glycoprobe for light-controlled photo-switchable cell imaging and photo-activated target recognition, resulting in an increased sensing precision.

## Introduction

Optical imaging offers great opportunities to monitor and analyze disease-related metabolites in live cells with a high spatiotemporal resolution^1–5^. Recent advances achieved in the development of fluorescence imaging techniques and biosensing systems allow for the precise and rapid detection of intracellular species for biomedical applications^6–9^. Ideally, fluorescence probes are designed to selectively recognize a target species in complicated biological systems. However, determination of a given analyte in a realistic biological milieu (such as in cells and in vivo) with fluorescence is easily interfered by the complexity of microenvironment (e.g., change of pH and salt strength, and the existence of structurally complicated biomacromolecules) and the inevitable background signals^10–13^. In particular, fluorescence probes that rely on the emission intensity change (fluorimetric) are prone to being suffered from these issues. To circumvent this problem, several elegant approaches have been proposed including the ratiometric sensing rationale (emission shift upon selectively recognizing an analyte), which is currently a method of choice for biosensing and bioimaging^14–16^.

Photochromism, as another blossoming sensing rationale, has been increasingly employed for constructing smart sensors due to their unique ability to reversibly shift between two isomeric states in a remote light-controlled manner^17–19^. Upon an alternate irradiation of ultraviolet (UV) and visible (Vis) light, both the structure and reactivity of photochromores can be reversibly tuned, leading to diversity in design of small-molecular probes^20–28^. A “double-check” mechanism (i.e., the reversible in situ switch between the two isomeric states) can be conducted with photochromic probes to preclude false positive/negative signals. Whereas conventional sensing systems work through the passive interaction with an analyte, we envision that photochromism can be used to elicit probe reactivity before analyte binding^29^. For example, light can convert photochromic probes form one isomeric state (inert to analyte) to the other (reactive to analyte) in situ, leading to sensing precision in a remote-controlled manner. In addition, the remote light-control over photochromic probes is mild and non-invasive with high spatiotemporal resolution.

**Fig. 1:**
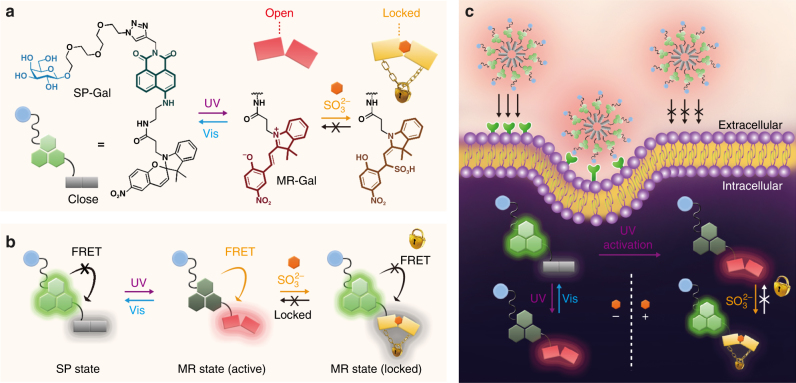
Molecular design of the photochromic fluorescent SP-Gal and its working mechanism in solution and intracellularly. **a** Structure and photochromism/sulfite (SO_3_^2−^) reactivity of SP-Gal. **b** Schematic illustration of the fluorescence “off-on” switching (FRET = Förster Resonance Energy Transfer) and sulfite-induced fluorescence locking of SP-Gal. **c** Schematic illustration of receptor-targeting intracellular photochromism of glycoprobe and their target (sulfite) recognition controlled by remote light

To prove our hypothesis, herein we developed photochromic fluorescent glycoprobes for remote light-controlled recognition of a target intracellular species produced both exo- and endogenously. Shown in Fig. 1a is the principle by which to design the photochromic glycoprobes. We first coupled a fluorophore (naphthalimide that produces fluorescence signal) with a spiropyran (SP), which can be reversibly light-converted to merocyanine (MR). Then, the resulting conjugate was equipped with a glycoligand (D-galactose (Gal)), producing SP-Gal to selectively target a transmembrane glycoprotein receptor. After alternate UV/Vis irradiations, the photochromic probes reversibly switched between the SP (close) and the MR (open) state. The photoisomerization of the probe was interpreted by naphthalimide fluorescence, which is reversibly switched (“off/on” via “UV/Vis-light” irradiation) by Förster resonance energy transfer (FRET) from naphthalimide to MR rather than SP (Fig. 1b). A fluorescence “blinking” effect could be observed upon the photo-controlled off–on cycles for precise imaging. Besides, the remote light-“activated” MR state can subsequently react with a target species (sulfite ion [SO_3_^2−^]), producing a “locked state” in which the fluorescence remains constantly “on”. We further show that the amphiphilic glycoprobes, which form micelles in water, can assemble in live cells that express selective Gal receptors (Fig. 1c). The selectively endocytosed glycoprobes have also proven to be amenable for photochromic cycling in a specific organelle as well as remote light-controlled recognition of intracellular sulfite.

## Results

### Photochromism and sulfite detection of SP-Gal in buffer solution

The SP-Gal (Fig. 1a and Supplementary Fig. 18) and a control compound SP-PEG (Supplementary Fig. 19) with a polyethylene glycol (PEG), instead of Gal, were synthesized. SP is a popular photochromic molecule, which can be light-converted to the charge-separated zwitterionic MR structure after photoisomerization^19, 25, 29^. A variety of photochromic probes have been constructed based on SP since the zwitterionic isomer (MR) offers a coordination site for various analytes, such as ions and biomolecules^30–32^. In addition, photoisomerization of SP produces the MR isomer with an extended conjugated system, which can serve as a FRET acceptor to tune the fluorescence of closely coupled fluorophores^33, 34^. The double bond of the hemicyanine-like motif of MR is also prone to undergoing Michael addition reactions with nucleophiles, making possible the development of reaction-based probes^35–37^. Bearing these points in mind, the glycoprobes were synthesized through a simple coupling of naphthalimide with SP, producing SP-Gal in good yields. The fluorophore (naphthalimide) was coupled with D-galactose to target a specific transmembrane glycoprotein receptor, by a click reaction. The control compound (SP-PEG) was synthesized in a similar way.

**Fig. 2:**
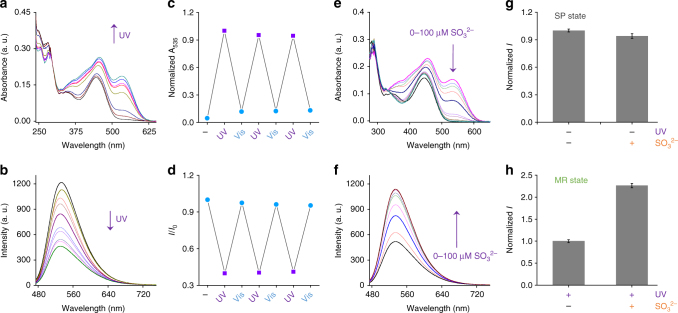
Spectroscopic analyses of photochromic SP-Gal and the remote light-controlled fluorescence sensing of sulfite. **a** UV–Vis absorbance spectral changes of SP-Gal (10 μM) in phosphate buffered saline (PBS, 0.01 M, 1% DMSO, pH 7.4) at 298 K upon irradiation at 365 nm (2.6 mW cm^−2^). **b** The fluorescence spectral changes of SP-Gal (10 μM) in PBS (0.01 M, 1% DMSO, pH 7.4) at 298 K upon irradiation at 365 nm (2.6 mW cm^−2^). **c** UV-Vis absorbance photo-switching (at 535 nm) of SP-Gal/MR-Gal (10^−5^ M) in PBS (0.01 M, 1% DMSO, pH 7.4) at 298 K. **d** Fluorescence emission photo-switching (at 544 nm; excitation wavelength: 450 nm) of SP-Gal/MR-Gal (10^−5^ M) in PBS (0.01 M, 1% DMSO, pH 7.4) at 298 K, where I_0_ and I are the initial fluorescence intensity of SP-Gal and that of the corresponding SP-Gal**/**MR-Gal upon alternate UV–Vis irradiation, respectively. **e** The UV-Vis absorbance change of MR-Gal (10 μM, in PBS buffer) with SO_3_^2−^ (from 0 to 100 μM). **f** The fluorescence change of MR-Gal (10 μM, in PBS buffer) with SO_3_^2−^ (from 0 to 100 μM). **g**, Fluorescence intensity ratio of SP-Gal without UV irradiation (365 nm) in the absence and presence of SO_3_^2−^ (100 μM). **h** Fluorescence intensity ratio of MR-Gal (SP-Gal with UV irradiation at 365 nm) in the absence and presence of SO_3_^2−^ (100 μM). The error bar represents SD (*n* = 3)

With the probes in hand, their photochromism was first tested in an aqueous buffer solution by UV–Vis absorbance and fluorescence spectroscopy (Fig. 2 and Supplementary Fig. 1). After irradiation with UV light (365 nm), a gradually enhanced absorbance peak of SP-Gal at 535 nm was observed, characteristic of the formation of the MR-Gal isomer (Fig. 2a). The peak remained unchanged after 7 min of irradiation, suggesting that the probe reached a photostationary state. In parallel, the fluorescence of SP-Gal at 532 nm decreased sharply by *ca*. 65% after UV irradiation (Fig. 2b) because of FRET from naphthalimide donor to the MR acceptor^33, 34^. Then, we determined that the fluorescence was recovered after a Vis light irradiation (530 nm for 5 min), which we ascribe to the reversible isomerization from the MR state back to the initial SP state. The FRET efficiency of MR-Gal was calculated to be 65.9% (*E* = 1−*τ*/*τ*_0_, Supplementary Table 1). The light-controlled reversible absorbance (Fig. 2c) and fluorescence switch (Fig. 2d) could be realized several cycles without obvious degradation in intensity. The kinetics of photo-switching of both directions (k_sp-mr_ = 0.024 s^−1^; k_mr-sp_ = 0.031 s^−1^; 2.6 mW cm^−2^ for UV irradiation at 365 nm and 150 mW for visible light at 530 nm, Supplementary Fig. 2) and the photochromic quantum yields (Φ_sp-mr_ = 5.78 ± 0.1%; Φ_mr-sp_ = 14.41 ± 0.1%; the error bar represents s.d. (*n* = 3), Supplementary Table 1) in aqueous solution were also determined.

Then, the light-controlled recognition of SP-Gal for a nucleophile was carried out in solution. Sulfite ion (SO_3_^2−^) is generated endogenously from L-cysteine and thiosulphate, playing an important role in biological sulphur cycles in the human body^38^. However, the existence of sulfite ions in the body fluid above normal concentrations is linked to several human diseases^39^. Considering their nucleophilic nature, Michael addition of sulfite to the hemicyanine moiety of MR-Gal may break the π-conjugation, thereby changing the optical properties of the probe. Furthermore, unlike common Michael addition probes, hemicyanine is inert to biothiols (e.g., cysteine, homocysteine, and glutathione), which can interfere with sulfite sensing in a realistic biological environment^37^. We first determined that SP-Gal without UV irradiation was not reactive to sulfite (Fig. 2g) and a range of other biothiols (Supplementary Fig. 3a, b), suggesting that the SP state is not reactive with the target analyte. Then, SP-Gal was photo-isomerized (UV activation) to the MR-Gal state prior to sulfite addition. We observed that the characteristic merocyanine absorption band around 535 nm was gradually decreased after adding increasing sulfite (Fig. 2e and Supplementary Fig. 3e), while the fluorescence of the MR-Gal was simultaneously increased (Fig. 2f, h and Supplementary Fig. 3e). After treatment of the probe with sulfite, however, the resulting fluorescence remained constantly “on” irrespective of the alternate UV/Vis irradiation (Supplementary Fig. 3c; the absorbance spectrum also hardly changed under the same condition as shown in Supplementary Fig. 3d), suggesting the formation of the Michael adduct between MR-Gal and sulfite (Fig. 1a). ^1^H NMR and mass titration clearly showed the generation of a new compound MR-Gal**-**SO_3_^2−^after treatment of MR-Gal with sulfite (Supplementary Fig. 4). Kinetics studies on MR-Gal with sulfite (Supplementary Fig. 5) suggest a first-order reaction kinetics between MR-Gal and sulfite ion, which is in accordance with previous reports on Michael addition based sulfite detection with hemicyanine derivatives^37^. As a consequence, the “constantly on” fluorescence was probably caused by the formation of the Michael adduct, breaking the π-conjugation of hemicyanine, and thus locking the FRET (Fig. 1b).

### Targeted intracellular imaging and photochromism of SP-Gal

Having tested the light-controlled optical performances of SP-Gal in solution, the ability of the glycoprobes for targeted intracellular photochromism was tested. A human hepatoma cell line (Hep-G2) that highly expresses the asialoglycoprotein receptor (ASGPr, selective for Gal) and two control cell lines (human cervical cancer (HeLa) and human lung cancer (A549)) without ASGPr expression were used (Supplementary Fig. 6d)^40, 41^. The cells were treated with SP-Gal by using SP-PEG as a control. We determined that SP-Gal selectively imaged Hep-G2 rather than A549 and HeLa cells (Supplementary Fig. 6a, b), whereas the fluorescence of SP-PEG without the “Gal-antenna” was distributed in all the cells used (Supplementary Fig. 6a and c). This suggests the good specificity of SP-Gal equipped with a Gal “warhead” to actively target the transmembrane receptor, and that the presence of Gal largely diminishes the unselective cell internalization of the glycoprobes. As shown in Fig. 1, SP-Gal incorporates a hydrophilic PEG linker between the Gal-ligand and the hydrophobic fluorophore/photochromore conjugate. This facilitates the formation of amphiphilic micelles of SP-Gal in sub-micrometer size, which is determined by both dynamic light scattering and transmission electron microscopy (Supplementary Fig. 7a–d). The formation of amphiphilic micelles may increase their binding avidity for receptor proteins through multivalent carbohydrate-receptor interactions^42^, and the hydrophilic galactosyl “shell” may also protect the glycoprobes from being unselectively internalized by cells without receptor expression^40^.

To prove the advantage of the presence of the PEG linkage, we also synthesized a control glycoprobe with just a rigid triazole linker that connects between the hydrophilic Gal and the hydrophobic dye conjugate (Supplementary Fig. 20). We determined that this compound exhibited an anomalous morphology (Supplementary Fig. 7e–h) and showed a much worse effect in selective cell imaging than SP-Gal (Supplementary Fig. 8), suggesting the importance of the molecular design for amphiphilic glycoprobes. To further prove the ASGPr-targeting ability of SP-Gal, we used a sh-ASGPr cell line with a reduced ASGPr expression level by shRNA transfection (Supplementary Fig. 9c)^41^. We determined that the SP-Gal fluorescence was largely decreased in sh-ASGPr with respect to Hep-G2 (Supplementary Fig. 9a, b). In the meanwhile, pretreatment of Hep-G2 with increasing free D-galactose led to a gradual suppression of the fluorescence of SP-Gal, suggesting that the selective fluorescence imaging is based on Gal-ASGPr recognition (Supplementary Fig. 9c, d). SP-Gal was determined to be barely toxic to Hep-G2, even with a concentration 8-fold higher than that used for imaging (Supplementary Fig. 10a).

**Fig. 3:**
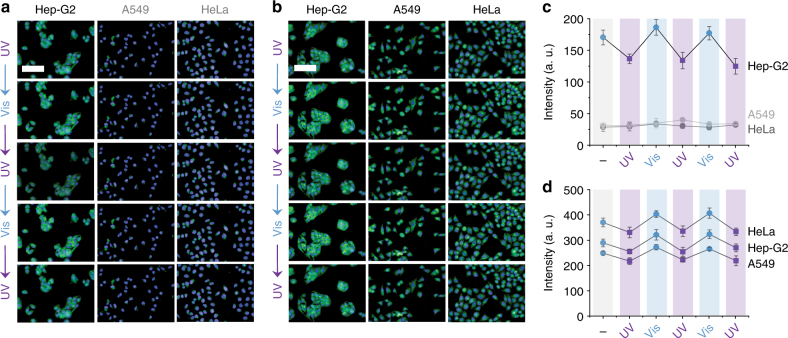
Receptor-targeting intracellular photochromism of glycoprobes by UV/Vis cycling. **a** UV/Vis cycling of SP-Gal (20 μM) in different cells (Hep-G2 = human hepatoma cell line; HeLa = human cervical cell line; A549 = human lung cell line). **b** UV/Vis cycling of SP-PEG (20 μM) in different cells. **c**, Fluorescence quantification of UV/Vis cycling of SP-Gal in different cells. **d** Fluorescence quantification of UV/Vis cycling of SP-PEG in different cells. For fluorescence imaging, the excitation wavelength was 360–400 nm and 440 nm and emission channel 410–480 nm and 450–550 nm for Hoechst and SP-Gal/SP-PEG, respectively (scale bar: 100 μm, which is applicable to all images; The error bar represents s.d. (*n* = 3))

Next, we set out to test the targeted intracellular photochromism of the glycoprobes controlled by remote light. We alternately irradiated the cell lines pretreated with SP-Gal or SP-PEG by UV/Vis (365/530 nm) light, and then recorded the resulting fluorescence intensity (Fig. 3). We observed a clear fluorescence decrease/increase cycle upon UV/Vis irradiation of SP-Gal/MR-Gal for Hep-G2 rather than HeLa and A549 (Fig. 3a, c). In contrast, the photochromic switch of SP-PEG/MR-PEG was observed in all the cell lines tested (Fig. 3b, d). This suggests that, in addition to being selectively internalized, the photochromic glycoprobes are capable of taking remote light orders for intracellular photochromic actions. To preclude the damaging effect of the light used, the cell viability was checked under alternate UV/Vis irradiations. We determined that the cells with or without the photochromic glycoprobes were perfectly viable for up to six cycles of photo-irradiation (Supplementary Fig. 10b), demonstrating a low photo-toxicity of our photo-switching procedure.

### Photo-switchable imaging of lysosomes with SP-Gal

**Fig. 4:**
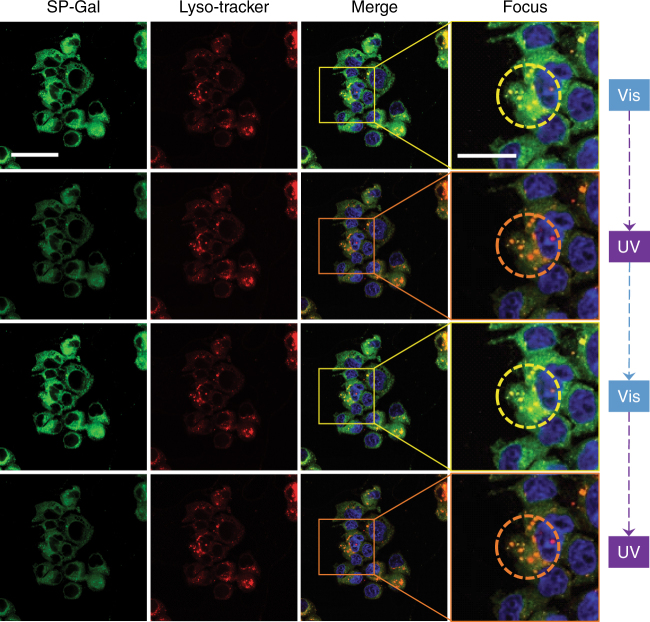
UV/Vis cycling and co-localization of photochromic glycoprobes with lysosome tracker in Hep-G2 cells. The excitation wavelength was 405, 440, and 577 nm and emission channel 420–460, 535, and 590 nm for Hoechst, SP-Gal and Lyso-tracker Red, respectively (the “Focus” column represents an enlarged area of the “Merge” column as framed; the circles in the “Focus” column highlight the photochromic actions of SP-Gal in lysosomes). Scale bar for all images in the Sp-Gal, Lyso-tracker and Merge groups is 40 μm. Scale bar for images in the “Focus” group is 20 μm

Since ASGPr is responsible for trafficking endocytosed species to the lysosome, we also used confocal laser scanning microscopy to track the photochromic glycoprobes. Considering the acidic nature of lysosome (pH 4.5–5.5), the photo-switching behavior of SP-Gal was first examined under buffer solutions with different pH (from pH 3.0 to 7.4). The results showed that the photochromic actions were hardly influenced within this acidic pH range (Supplementary Fig. 11). In addition, we tested the photo-switching of our SP-Gal probe intracellularly with different pH. We observed that the photo-switching of SP-Gal similarly functioned well with an intracellular pH range of 4.0–7.0 (Supplementary Fig. 12), which covers the pH range of lysosome. With these promising results in hand, co-localization of SP-Gal in lysosome was carried out. Co-localization of SP-Gal fluorescence with that of a lyso-tracker showed that the photochromic glycoprobes were largely assembled in lysosome (Fig. 4, Merge; Pearson’s correlation coefficient: 0.76). A subsequent UV/Vis light irradiation assay showed that the SP-Gal fluorescence, as localized in lysosome, could be tuned off (UV to MR-Gal) and on (Vis back to SP-Gal) repeatedly (Fig. 4 (Focus) and Supplementary Fig. 13).

### Photo-activated detection of exogenous intracellular sulfite

**Fig. 5:**
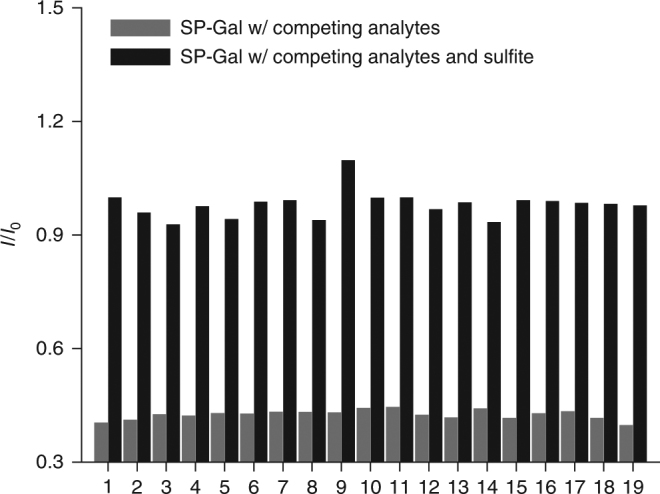
Biospecificity of photochromic fluorescent glycoprobe. The fluorescence change of MR-Gal (10^–5^ M) with a range of competing analytes (10^−3^ M) and the mixture of the analytes (10^−3^ M) with sulfite (5 × 10^−5^ M) in phosphate buffered saline (PBS, 0.01 M, 1% DMSO, pH 7.4) at 298 K (excitation wavelength: 450 nm), where I_0_ and I are the fluorescence of the initial spiropyran state and that of the UV-activated merocyanine state, respectively. Competing analytes 1–19: 1: Blank (probe alone), 2: F^−^; 3: Cl^−^; 4: Br^−^; 5: I^−^; 6: NO_3_^−^; 7: NO_2_^−^; 8: CH_3_COO^−^; 9: HCO_3_^−^; 10: SO_4_^2−^; 11: S_2_O_3_^2−^; 12: PO_4_^3−^; 13: CO_3_^2−^; 14: Cys; 15: Hcy; 16: GSH; 17: HPO_4_^2−^; 18: H_2_PO_4_^−^; 19: N_3_. We note that the physiologic concentration of common thiols and nucleophiles such as Hcy, Cys is in the range of 10–200 μM^49–51^, which is well-below the concentration used in this experiment

Eventually, as a proof-of-concept, the remote light-controlled conversion of SP-Gal to MR-Gal for sulfite recognition was carried out in Hep-G2 cells. We first tested the biospecificity of MR-Gal for sulfite with a variety of other metabolic nucleophiles. The result showed that the MR-Gal fluorescence was barely changed by unselective thiols and nucleophile species with concentrations above their physiologic levels (Fig. 5). The sequential addition of 5 equiv. of sulfite significantly recovered the probe fluorescence, demonstrating the specificity in reactivity of merocyanine for sulfite (Fig. 5).

**Fig. 6:**
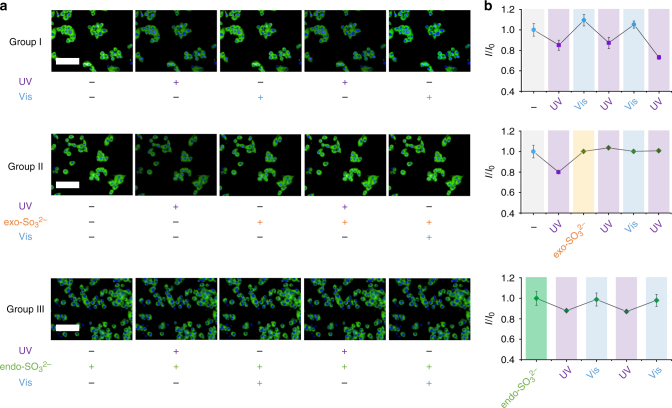
Intracellular, remote light-controlled recognition of sulfite by photochromic glycoprobes in Hep-G2 cells. **a** Fluorescence imaging of SP-Gal (20 μM, Group I), SP-Gal (20 μM) with exogenous (exo-) sulfite (80 μM, Group II) and SP-Gal (20 μM) with endogenous (endo-) sulfite (pretreating the cells with 400 ng mL^−1^ lipopolysaccharide, Group III) upon alternate UV/Vis irradiations. **b** Fluorescence quantification of SP-Gal (Group I), SP-Gal with exogenous sulfite (Group II) and SP-Gal with endogenous sulfite (Group III) upon alternate UV/Vis irradiations, where I_0_ and I are the initial fluorescence intensity of SP-Gal and that of the corresponding SP-Gal/MR-Gal/MR-Gal-SO_3_^2−^ state upon photo-cycling and sulfite reaction, respectively. For fluorescence imaging, the excitation wavelength was 360–400 nm and 440 nm and emission channel 410–480 and 450–550 nm for Hoechst and SP-Gal/SP-PEG, respectively (scale bar: 100 μm, which is applicable to all images; The error bar represents s.d. (*n* = 3))

SP-Gal was first internalized by Hep-G2, and then the cells were treated with UV irradiation for converting SP to the MR state. We determined that, after addition of exogenous sulfite (by treatment of cells with sodium sulfite), the decreased fluorescence of MR-Gal increased spontaneously without visible irradiation (Fig. 6, Group II). Notably, the increased fluorescence of MR-Gal could not be further suppressed by UV irradiation, leading to a constantly “on” fluorescence of the probe irrespective of alternate light irradiations (Fig. 6, Group II). This phenomenon is contrary to that observed for cells without sulfite, for which the UV/Vis controlled reversible SP-Gal/MR-Gal photoisomerization was available (Fig. 6, Group I). The locked fluorescence might suggest the reaction of sulfite with the probe at the MR state, as similarly shown by the solution-based experiments (Fig. 2f, g), producing the “locked” MR-Gal derivative (i.e., the Michael adduct). A subsequent analysis by high-performance liquid chromatography showed that the Michael adduct of MR-Gal with sulfite was traced in the lysate of Hep-G2 pretreated sequentially with SP-Gal, UV irradiation and then SO_3_^2−^ (Supplementary Fig. 14). This corroborates that the irreversible fluorescence enhancement was a result of the addition of sulfite to MR-Gal in live cells, thereby demonstrating the amenability of the photochromic glycoprobes for remote light-controlled recognition of intracellular sulfite ions.

To further test the intracellular stability of SP-Gal, we kept Hep-G2 cells preincubated with SP-Gal in dark for 30 min with or without the presence of sulfite (Supplementary Fig. 15). We observed that the intracellular fluorescence of SP-Gal barely changed with time (up to 30 min) in dark, irrespective of the presence of sulfite. A subsequent UV irradiation of the cells without sulfite generated MR-Gal, which was able to undergo the photochromic cycling through alternate UV/Vis treatment (Supplementary Fig. 15a, b). However, UV irradiation of the cells with sulfite led to a quick fluorescence increase spontaneously, and then the probe became insensitive to light orders (Supplementary Fig. 15c, d). These data suggest that the photochromic glycoprobes can be kept inert (the SP-Gal state) intracellularly even in the presence of the target molecule.

### Photo-activated detection of endogenous intracellular sulfite

Next, we tested whether the probe could react with endogenous sulfite by pretreating the cells with lipopolysaccharide (LPS), which can induce the inflammatory response of cells to produce low-level sulfite endogenously (Fig. 6, Group III)^43^. We observed that the photochromic fluorescence blinking of SP-Gal/MR-Gal was much weakened in cells with endogenous sulfite produced by LPS. By a fluorescence calibration with MR-Gal (Supplementary Fig. 16a), we determined that the sulfite concentration produced by LPS simulation in the lysate of Hep-G2 was 0.865 μM, which approximates that obtained by ion chromatography (0.70 μM, Supplementary Fig. 16c). To further demonstrate the sensitivity of our probe, the quantification of LPS-stimulated endogenous sulfite was also carried out with live cells. By a fluorescence calibration with MR-Gal (Supplementary Fig. 16b), we determined that the sulfite ion concentration produced by LPS simulation in live Hep-G2 cells was 0.904 μM, which approximates that obtained in cell lysate and by ion chromatography. The limit of detection of our probe was checked under different concentration ranges (Supplementary Fig. 17), and the results showed a good linearity, which is within the reported sulfite level found in the human serum and rat liver^44–46^. More importantly, the probe could respond to a slight endogenous increase of sulfite concentration (below 1 μM) through LPS simulation of Hep-G2 cells (Fig. 6, Group III). These results clearly suggest that the photochromic glycoprobe developed can sensitively detect an abnormally elevated sulfite level in a remote light-controlled manner.

To summarize, we have developed here a unique, remote light-controlled fluorescent glycoprobe for sulfite ion based on the finely tunable photochromism. The probe is equipped with an antenna (galactose) to actively localize a cell with a selective receptor. The selectively endocytosed probes can thereby operate remote, alternate UV/Vis light orders intracellularly to reversibly switch between two photoisomeric states, outputting a dynamic, “blinking” fluorescence signal. Further, the remote light conversion of the photochromic probe from one isomeric state to the other activated its reactivity towards sulfite ion elicited both exo- and endogenously, locking the fluorescence. This research sets the basis for the development of a new generation of smart diagnostic probes based on photochromic principles. Development of photochromic probes for a broader range of other biologically and pathologically important species is currently underway in our laboratories.

## Methods

### Materials and instruments

Chemicals were used as received unless otherwise indicated. All oxygen or moisture-sensitive reactions were performed under argon atmosphere using the standard Schlenk method. All other reagents are of analytical purity and used without further purification. Solvents used are of analytical grade, except those for recrystallization and optical tests, which were distilled prior to use. Thin-layer chromatography (TLC) was carried out on aluminum sheets coated with silica gel 60 F254 (MERCK).^1^H NMR and^13^C NMR spectra were recorded using Bruker AM-400 spectrometers. DMSO-*d*_6_, CDCl_3_ and D_2_O were used as solvent. Absorption and fluorescence spectra were recorded using Varian Cary 500 and Varian Cary Eclipse, respectively. The UV (365 nm, 2.6 mW cm^−2^) and light-emitting diode (LED) lamps M530L2 (Thorlabs; Norminal Wavelength 530 nm, Bandwidth (FWHM) 33 nm, 150 mW) were used as light sources for UV and visible light irradiation, respectively. Hep-G2 (HB-8065^TM^), HeLa (CCL-2^TM^) and A549 (CCL-185^TM^) were obtained from ATCC (American Type Culture Collection).

### Photochromic kinetics in solution

Stock solutions of SP-Gal, SP-Gal 2 and SP-PEG (1 mM) were prepared in DMSO. Test solutions of SP-Gal, SP-Gal 2 and SP-PEG (10 μM) were prepared in phosphate buffered saline (PBS, 0.01 M, pH 7.4, 1% DMSO). The light-controlled reversible fluorescence switches for glycoprobes were carried out with a path length of 5 mm and an excitation wavelength at 450 nm by scanning the spectra between 470 and 750 nm. The bandwidth for both excitation and emission spectra was 5 nm. Unless otherwise mentioned, all the spectra were recorded at 298 K. For a photochromic cycling, the solution was irradiated with UV light (365 nm, 2.6 mW cm^−2^) in darkroom, and the fluorescence spectroscopy was tested every 30 s until the peak retained unchanged. Then, the solution was reversibly irradiated with Vis light (530 nm, 150 mW) in darkroom, and the fluorescence spectroscopy was tested every 30 s until the peak retained unchanged.

### Calculation of photochromic and fluorescence quantum yields

The fluorescence quantum yields of SP-Gal**/**MR-Gal were measured by Quanta-w F-3029 Integrating Sphere. The photochromic quantum yields were measured based on the following equation:^47^

$$\Phi = \frac{{m \times V \times h \times c \times {N_A}}}{{\lambda \times d \times {P_0} \times {\varepsilon _{{\rm{prod}}}} \times \left( {1 - {{10}^{ - {A_0}}}} \right)}}$$

where *m* is the slope of the linear fit from the time-dependent changes in absorption at 530 nm during irradiation at 365 nm (ring-opening) and 530 nm (ring-closing), respectively; *V* is sample volume, *h*×*c*×*N*_*A*_ is a constant, *P*_0_ is the irradiation intensity, *λ* is the excitation wavelength, *A*_0_ is the absorbance at excitation wavelength, *ɛ*_prod_ is the extinction coefficient and *d* is the cuvette thickness.

### Calculation of fluorescence lifetime and FRET efficiency

The fluorescence lifetime of SP-Gal**/**MR-Gal were measured by Edinburgh Lifespec-Ps spectrofluorometer (FL920). The energy transfer efficiency was calculated according to the following equation: *E* = 1−*τ*/*τ*_0_, where *E* is the energy transfer efficiency and *τ* and *τ*_0_ are the fluorescence lifetime of the donor (naphthalimide) with and without the energy acceptor (merocyanine), respectively^48^.

### Fluorescence spectroscopy for sulfite detection

Stock solutions of SP-Gal and SP-PEG (1 mM) were prepared in DMSO and then converted to MR-Gal and MR-PEG under UV light (365 nm, 2.6 mW cm^−2^), respectively. Stock solution of 1 mM of SO_3_^2−^ was prepared in PBS (0.01 M, pH 7.4). The fluorescence measurements for glycoprobes were carried out with a path length of 5 mm and an excitation wavelength at 450 nm by scanning the spectra between 470 and 750 nm. The bandwidth for both excitation and emission spectra was 5 nm. Unless otherwise mentioned, all the spectra were recorded at 298 K.

### Statistical analysis

Results are expressed as mean ± SD. Statistical analysis was performed with GraphPad PRISM (GraphPad Software, Inc.) using Student’s unpaired *t*-test. *P*-values <0.05 was considered statistically significant. All experiments were repeated at least three times with representative data shown.

### Cell culture

Hep-G2 and HeLa cells were maintained in a Dulbecco’s Modified Eagle’s Medium (Invitrogen, Carlsbad, CA, USA) supplemented with 10% fetal bovine serum (Gibco, Gland Island, NY, USA) and A549 cells were cultured in Ham’s F-12 nutrient mixture supplemented with 10% fetal bovine serum in a humidified atmosphere of 5% CO_2_ and 95% air at 37 °C.

### Cell viability assay

Cells were plated on 96-well plates in growth medium. After 24 h, cells were treated with SP-Gal of different concentrations for 15 min. Then, cells were gently washed with PBS once. After 72 h of incubation, 10 μL per well of MTS/PMS (20:1, Promega, Corp.) solution was added to each well, followed by a gentle shake. After incubation at 37 °C under 5% CO_2_ for 2 h, the absorbance of the solutions was measured at 490 nm, using an M5 microplate reader (Molecular Device, USA). The optical density of the result in MTS assay was directly proportional to the number of viable cells.

### Fluorescence imaging of cells

Cells were cultured in growth medium supplemented with 10% FBS. Then, cells (2.0 × 10^4^/well) were seeded on a black 96-well microplate with optically clear bottom (Greiner bio-one, Germany) overnight, and then incubated with 20 μM of glycoprobes for 20 min. After three rinses in PBS, the fluorescence was detected and photographed with an Operetta high content imaging system (Perkinelmer, USA).

### Establishment of the Hep-G2 knockdown stable cell line (sh-ASGPr)

Plasmids encoding ASGP-R1 specific shRNA was purchased from Santa Cruz Biotechnology, Inc. (Santa Cruz, CA, USA). Lentiviral particles were generated according to the manufacturer’s instructions. Briefly, cells were seeded in a six-well tissue culture plate and were grown to 80–90% confluency in antibiotic-free normal growth medium supplemented with FBS. Then, shRNA plasmid (3 μg) was cotransfected with pCAG-VSVG (1.8 μg) and PAX2 (2.7 μg) into cells using 15 μL of lipofectamine 2000 (Invitrogen, Carlsbad, CA, USA). After 6 h, the medium was changed to fresh DMEM with 10% FBS. After 72 h, the lentivirus-containing supernatant were collected, filtered, and then employed for analysis.

### Photochromic tests in cells

Cells were cultured in growth medium supplemented with 10% FBS. Then, cells (2.0 × 10^4^/well) were seeded on a black 96-well microplate with optically clear bottom (Greiner bio-one, Germany) overnight, and then were incubated with 20 μM of glycoprobes for 20 min. After three rinses in PBS the fluorescence was detected and photographed with an Operetta high content imaging system (Perkinelmer, US). To test the intracellular photochromic cycling, the 96-well microplate was irradiated with UV light (365 nm, 2.6 mW cm^−2^) in darkroom for 10 min, and then the fluorescence was detected and photographed with the Operetta high content imaging system. Subsequently, the 96-well microplate was reversibly irradiated with Vis light (530 nm, 150 mW) in darkroom for 10 min, and then the fluorescence was detected and photographed with the Operetta high content imaging system.

### Fluorescence spectroscopy for sulfite detection in cells

The probe was incubated with Hep-G2 cells w/ or w/o SO_3_^2−^ for 0 (−), 10, 20, and 30 min (in dark), and then was treated with UV irradiation. Following an incubation for another 10 min (in dark), the cells were treated with an alternate UV/Vis irradiation. For fluorescence imaging, the excitation wavelength was 360–400 nm and 440 nm and emission channel 410–480 nm and 450–550 nm for Hoechst and SP-Gal/SP-PEG, respectively. To elicit endogenous sulfite, cells (HepG2, 25000) were seeded on a black 96-well microplate with optically clear bottom (Greiner bio-one, Germany) and then treated with lipopolysaccharide (LPS, 80 ng mL^−1^) for 24 h.

### Confocal laser scanning microscopy

Hep-G2 cells were incubated sequentially with SP-Gal (40 μM, 1% DMSO in PBS, pH 7.4) and Lyso-Tracker Red (1 μM, 1% DMSO in PBS, pH 7.4) in an atmosphere of 5% CO_2_ and 95% air for 40 min at 37 °C. Then the cells on the microplate were rinsed by warm PBS and fixed by 4% paraformaldehyde for 20 min at room temperature. After rinsing twice with PBS, Hoechst 33342 (Invitrogen) (5 μg mL^−1^) was added to the cultures and incubated for 40 min at 37 °C. After three rinses with warm PBS, the fluorescence was detected and photographed with confocal laser scanning microscopy (Olympus, Japan).

### Data availability

The data that support the findings of this study are available from the corresponding author upon reasonable request.

## Electronic supplementary material

Supplementary Information
