# Supplementary material for: Remote light-controlled intracellular target recognition by photochromic fluorescent glycoprobes
Source: Nat Commun. 2017 Oct 17;8:987. doi: 10.1038/s41467-017-01137-8 (PMC5715093; doi:10.1038/s41467-017-01137-8)
Supplement: Supplementary file 1 — Supplementary Information [file 41467_2017_1137_MOESM1_ESM.pdf]

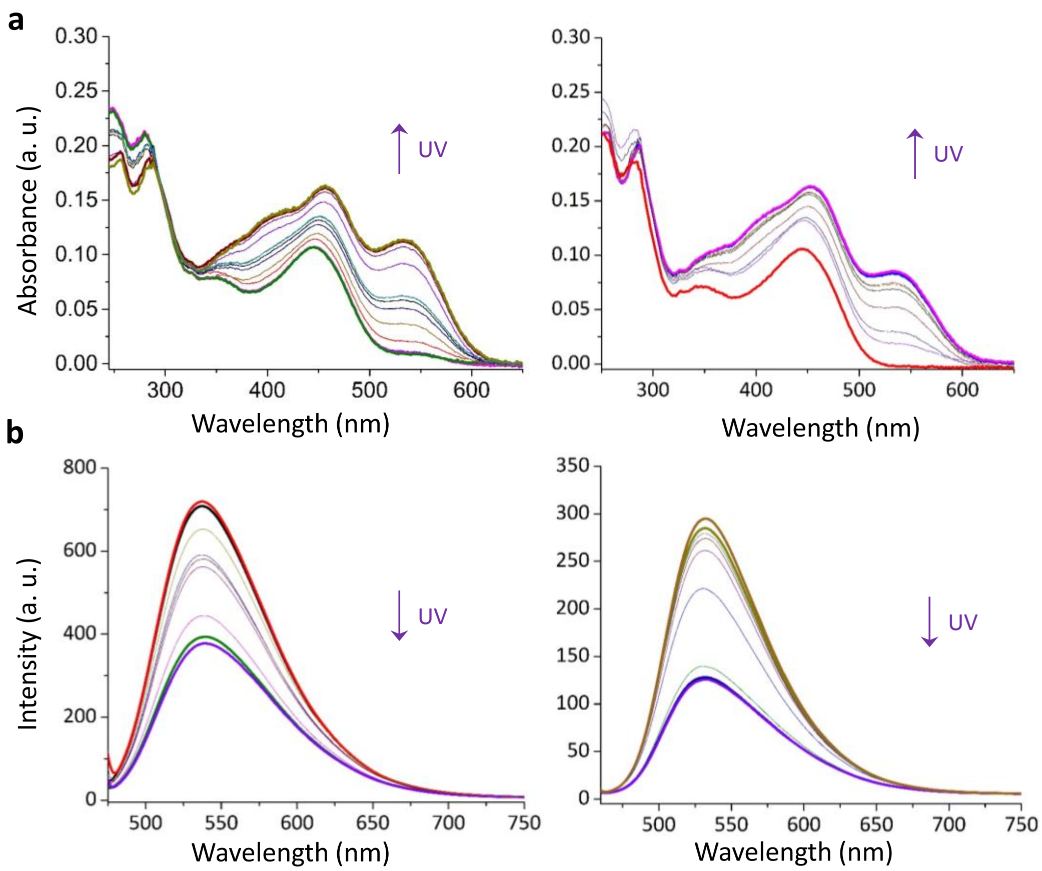

Supplementary Figure 1. UV-Vis absorbance spectral changes of (a) **SP-Gal** (left, 10 μM), and **SP-PEG** (right, 10 μM) in phosphate buffered saline (PBS, 0.01 M, 1% DMSO, pH 7.4) at 298 K upon irradiation at 365 nm (2.6 mW cm^{-2}). The fluorescence spectral changes of (b) **SP-Gal** (left, 10 μM), and **SP-PEG** (right, 10 μM) in PBS (PBS, 0.01 M, 1% DMSO, pH 7.4) at 298 K upon irradiation at 365 nm (2.6 mW cm^{-2}).

a

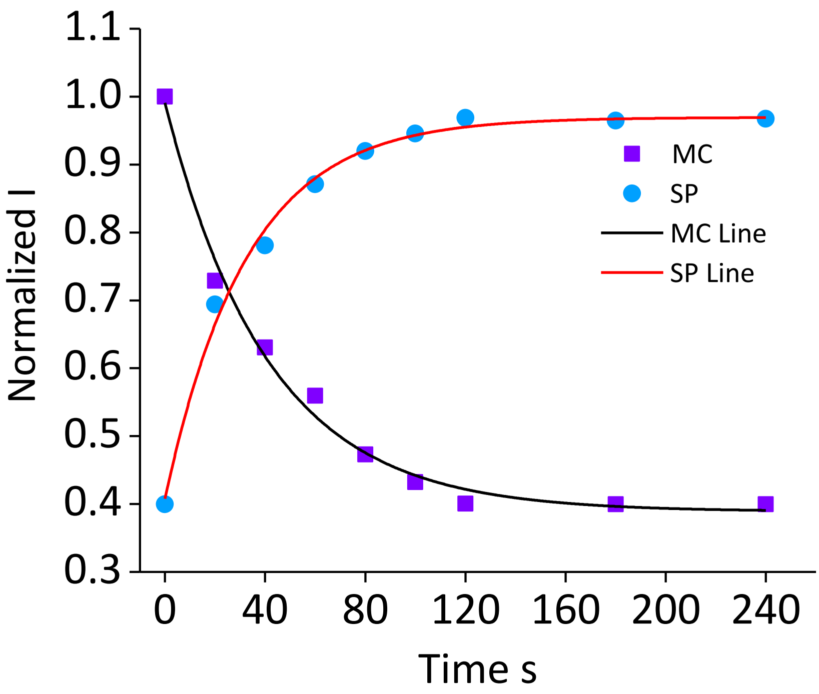

b

State	k (s^{-1})	R^2
SP-MR (black)	0.024	0.9889
MR-SP (red)	0.031	0.9919

Supplementary Figure 2. (a) Kinetic trace and (b) key parameters of ring opening (black line) and closing (red line) of **SP-Gal** in PBS (20 μM, 2.0 mL). The fluorescence intensity of the trace is normalized relative to the original fluorescence. Light intensity: 2.6 mW cm^{-2} for UV irradiation at 365 nm and 150 mW for visible light at 530 nm.

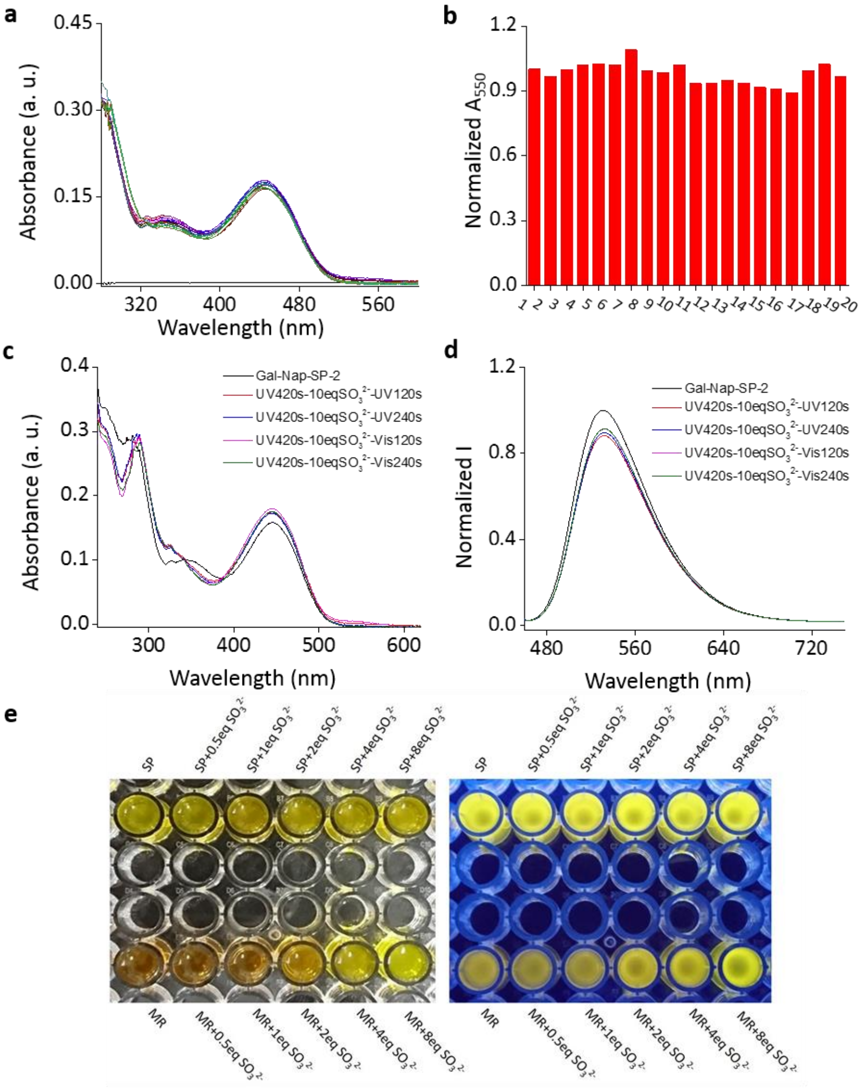

Supplementary Figure 3. (a) UV-Vis absorbance spectra of **SP-Gal** with various anions in PBS (pH 7.4, 1% DMSO, 0.01 M). (b) Fluorescence intensity change (I/I_0, where I and I_0 are the intensity with and without an analyte, respectively) of **SP-Gal** (10 μM) with various anions (Competing analytes 1-19: 1: Blank (probe alone), 2: F^-; 3: Cl^-; 4: Br^-; 5: I^-; 6: NO_3^-; 7: NO_2^-; 8: CH_3COO^-; 9: HCO_3^-; 10: SO_4^{2-}; 11: $S_2O_3^{2-}$; 12: PO_4^{3-}; 13: CO_3^{2-}; 14: Cys; 15: Hcy; 16: GSH; 17: HPO_4^{2-}; 18: $H_2PO_4^-$; 19: N^{3-}; 20: SO_3^{2-}) in PBS (pH 7.4, 1% DMSO, 0.01 M). (c) The UV-Vis absorbance change of **MR-Gal** (10 μM, in PBS) after addition of SO_3^{2-} (100 μM) and sequentially UV irradiation. (d) Fluorescence change of **MR-Gal** (10 μM, in PBS) after addition of SO_3^{2-} (100 μM) and sequentially UV irradiation. (e) Naked-eye colorimetric (left) and fluorescence (right; excited by a portable UV lamp at 365 nm, 2.6 mW cm^{-2}) of SO_3^{2-} in the presence of **SP-Gal/MR-Gal** (40 μM, 0.05 M PBS, 1% DMSO, pH 7.4).

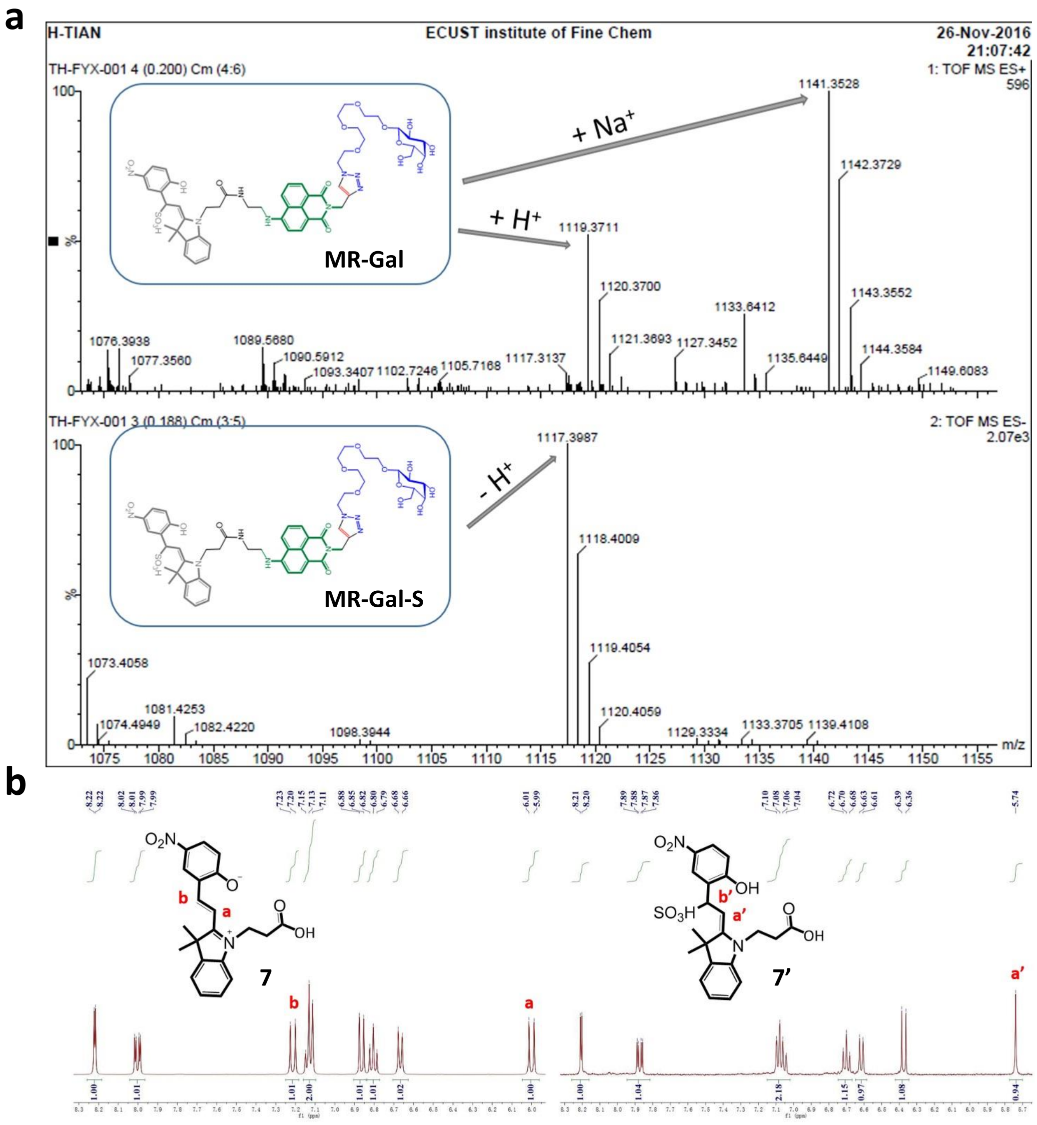

Supplementary Figure 4. (a) TOF-MS spectra of the resulting Michael adduct, **MR-Gal-SO_3^{2-}**, after treating **MR-Gal** with SO_3^{2-}. (b) Partial ^{1}H NMR spectrum of **Compound 7** and **Compound 7'** in the presence of SO_3^{2-} in DMSO-*d*6:D_2O = 1:4. Note: Because of the relatively low solubility of **MR-Gal** in DMSO-d_6/D_2O (the concentration for NMR test is much higher than in cells), we chose the corresponding merocyanine (photoisomer of spiropyran, **compound 7**) precursor of **MR-Gal** as the model molecule to test the Michael addition mechanism for sulfite detection. As shown below, after addition of 20 equivalents of sulfite to merocyanine (DMSO-d_6:D_2O = 1:4), an obvious up-shift of H_a (δ 6.0 to 5.7) was observed with the duplet split, converting to a singlet peak. More importantly, H_b (δ 6.87) on the reaction carbon site disappeared in the down-field area, indicating that the addition reaction has taken place in the speculated position.

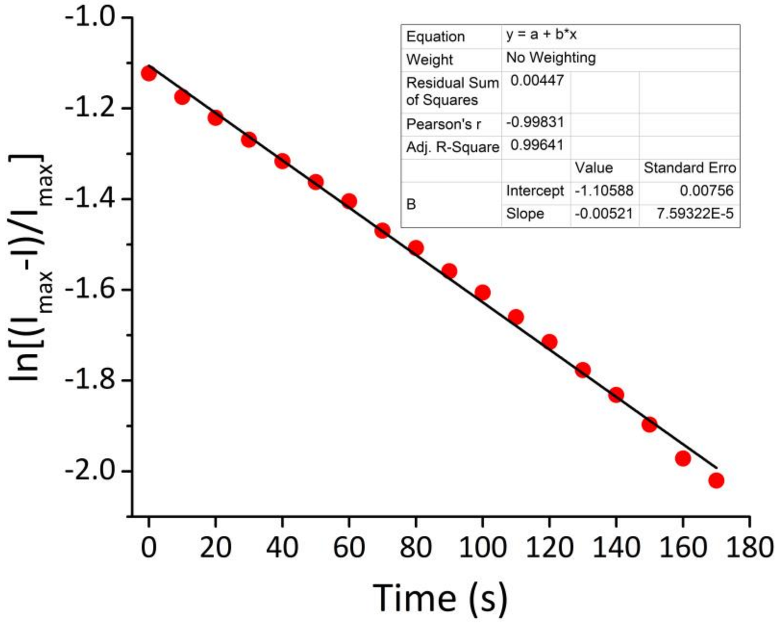

Supplementary Figure 5. **Plots of ln[(I_{max}-I)/I_{max}] as a function of time for the reaction of MR-Gal (20 μM in 0.05 M PBS, 1% DMSO, pH 7.4) with SO_3^{2-} (80 μM).** The time-dependent processes for Na_2SO_3 followed first-order kinetics with diverse observed rate constant k = 5.2×10^{-3} s^{-1}, R^2 = 0.996.

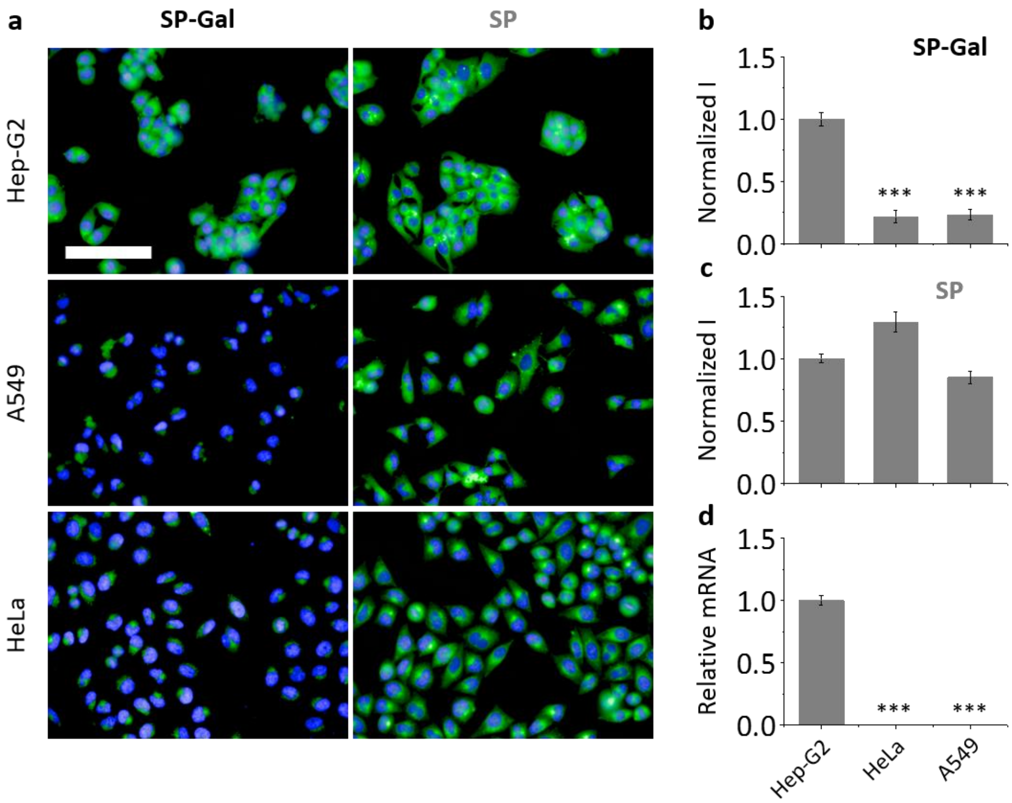

Supplementary Figure 6. **Receptor-targeting cell imaging of glycoprobes.** Fluorescence imaging (a) and quantification of (b) **SP-Gal** (20 μM) and (c) **SP-PEG** (20 μM) for different human cancer cell lines (Hep-G2 = human liver cancer; HeLa = human cervical cancer; A549 = human lung cancer). (d) Relative mRNA level of different cancer cells determined by real-time quantitative polymerase chain reaction (RT-qPCR) (***P < 0.001 with respect to Hep-G2); scale bar: 100 mm, which is applicable to all images. For fluorescence imaging, the excitation wavelength was 360-400 nm and 440 nm and emission channel 410-480 nm and 450-550 nm for Hoechst and **SP-Gal**/**SP-PEG**, respectively (scale bar: 100 μm, which is applicable to all images; the error bar represents s.d. (n = 3)).

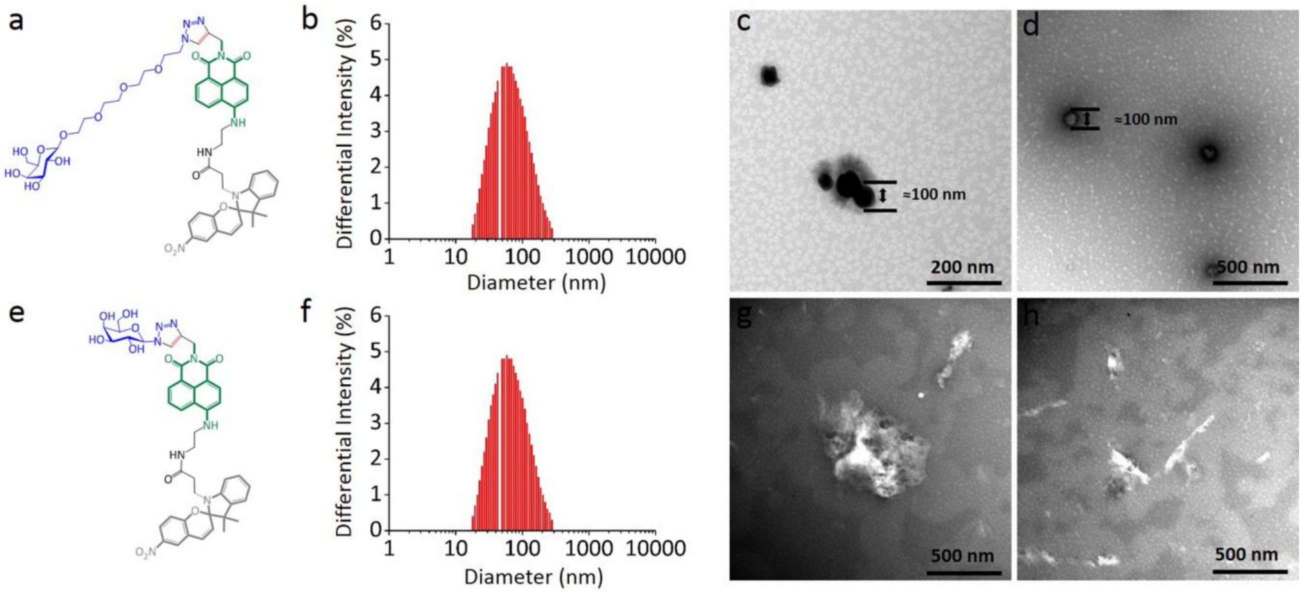

Supplementary Figure 7. Structure of (a) **SP-Gal** and (e) **SP-Gal 2**. Dynamic light scattering (DLS) of (b) **SP-Gal** (average size = 100 nm) and (f) **SP-Gal 2** (average size = 197 nm). The Transmission Electron Microscopic (TEM) images of (c, d) **SP-Gal** and (g, h) **SP-Gal 2**.

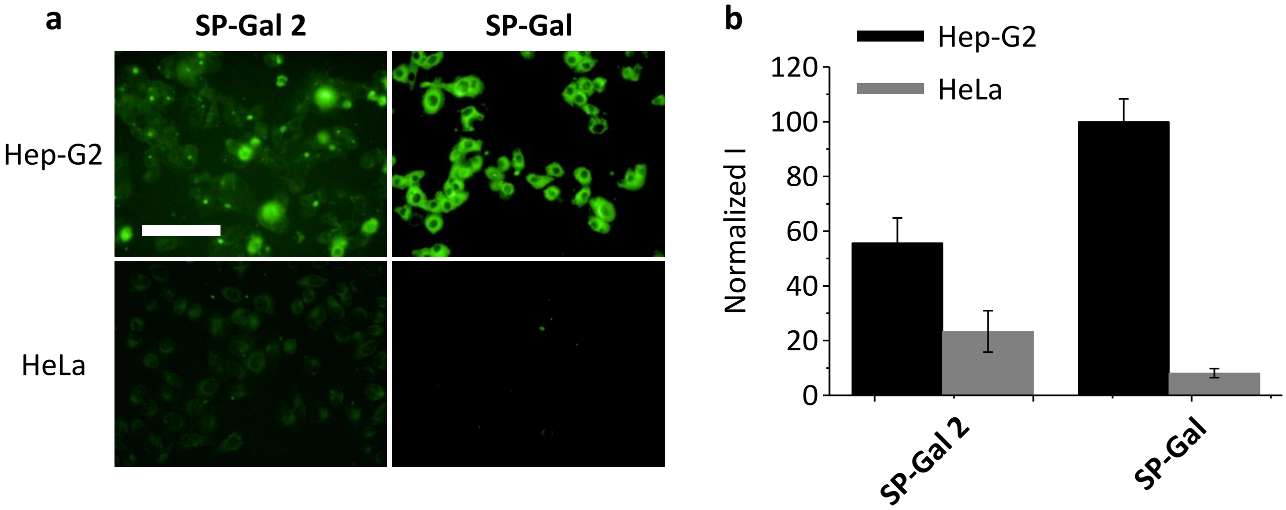

Supplementary Figure 8. (a) Fluorescence imaging of **SP-Gal 2** (20 µM) *vs.* **SP-Gal** (20 µM) for two different human cancer cell lines (Hep-G2 = human liver cancer; HeLa = human cervical cancer). (b) Fluorescence quantification of **SP-Gal 2** (20 µM) *vs.* **SP-Gal** (20 µM) for different cells. For all fluorescence images, the excitation wavelength was 440 nm and emission channel 450-550 nm (scale bar: 100 µm, which is applicable to all images; the error bar represents s.d. (n = 3)).

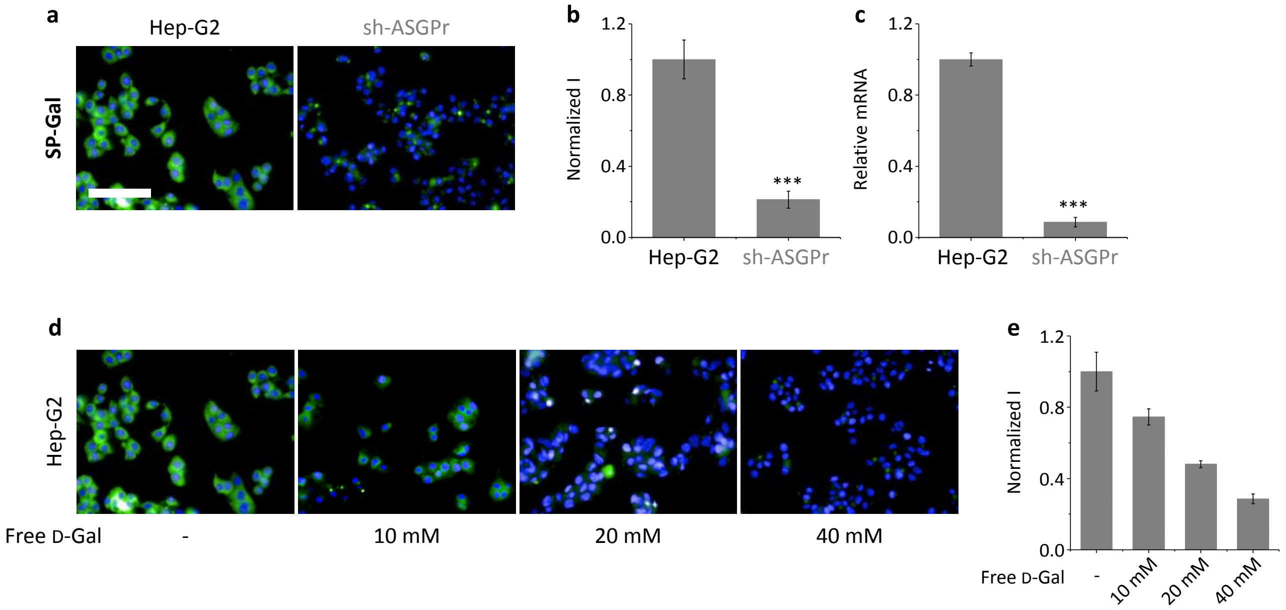

Supplementary Figure 9. Fluorescence imaging (a) and quantification (b) of **SP-Gal** (20 μM) for Hep-G2 with (sh-ASGPr) or without (control) knockdown of ASGPr (asialoglycoprotein receptor). (c) Relative mRNA level of sh-ASGPr and control determined by RT-qPCR (***P < 0.001 with respect to Hep-G2). Fluorescence imaging (d) and quantification (e) of **SP-Gal** (20 μM) for Hep-G2 cells pre-incubated with increasing galactose (D-Gal). For fluorescence imaging, the excitation wavelength was 360-400 nm and 440 nm and emission channel 410-480 nm and 450-550 nm for Hoechst and **SP-Gal**/**SP-PEG**, respectively (scale bar: 100 mm, which is applicable to all images; the error bar represents s.d. (n = 3)).

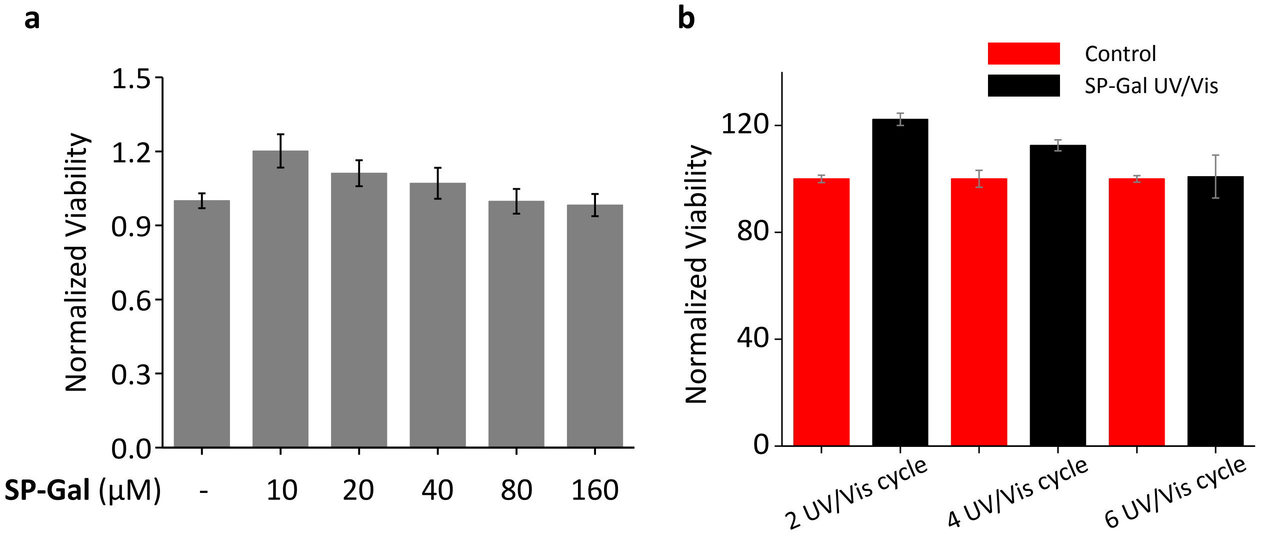

Supplementary Figure 10. (a) Viability of Hep-G2 in the presence of increasing **SP-Gal** determined by MTS assay. (b) Viability of Hep-G2 with and without **SP-Gal** under two, four and six alternate UV/Vis irradiation cycles determined by MTS assay. The error bar represents s.d. (n = 3).

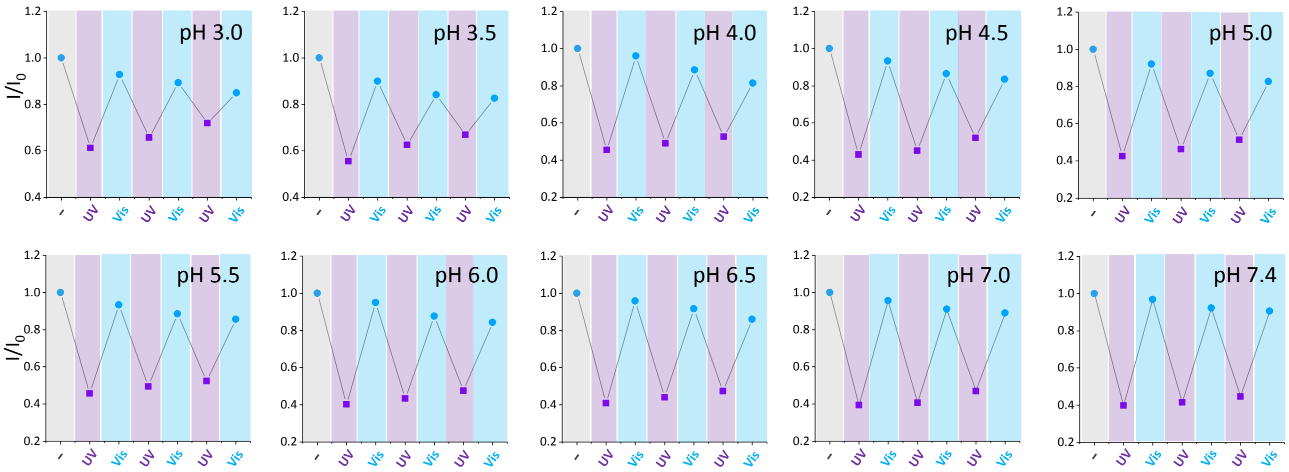

Supplementary Figure 11. **Photoswitching of SP-Gal with different pH.** Stock solution of **SP-Gal** (1 mM) was prepared in DMSO. Test solutions of **SP-Gal** (10 μM) were prepared in PBS (0.01 M, 1% DMSO) with different pH (3.0, 3.5, 4.0, 4.5, 5.0, 5.5, 6.0, 6.5, 7.0 and 7.4). I_0 and I are the initial fluorescence intensity of **SP-Gal** and that of the corresponding **SP-Gal/MR-Gal** upon alternate UV-Vis irradiation, respectively.

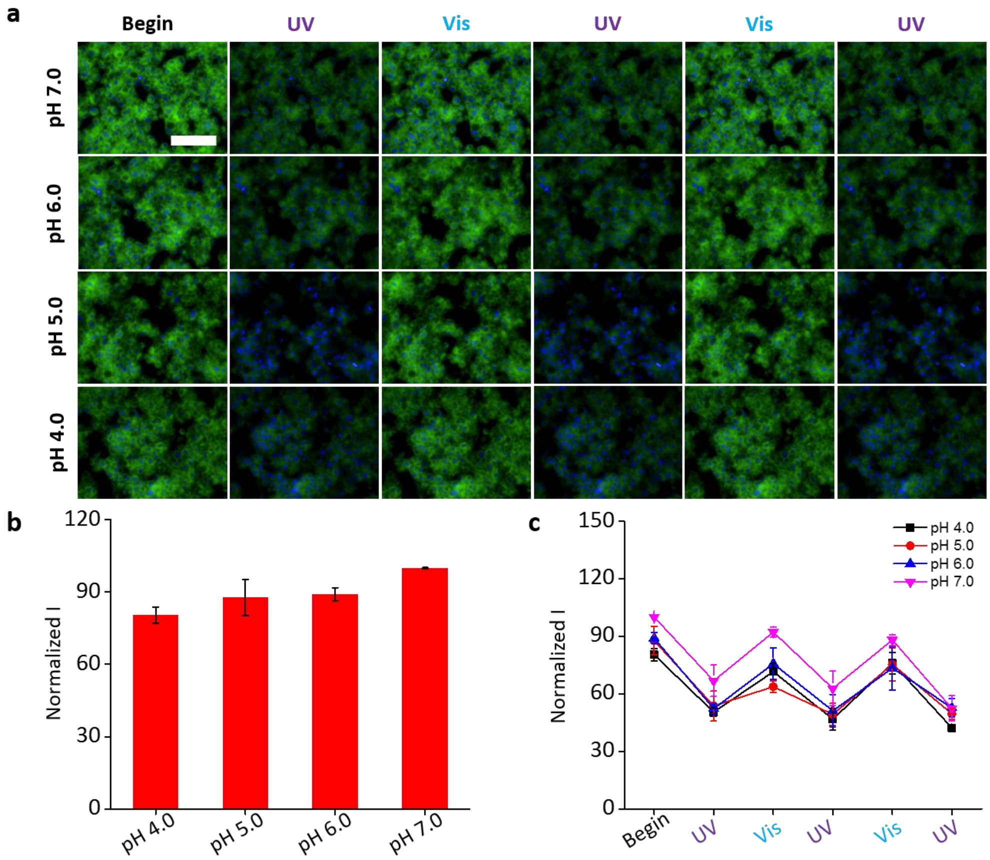

Supplementary Figure 12. (a) Fluorescence imaging of remote light-controlled photochromic cycling of **SP-Gal/MR-Gal** intracellularly with different pH. (b) Normalized fluorescence intensity of **SP-Gal** intracellularly with different pH. (c) Normalized fluorescence intensity of UV/Vis cycling of **SP-Gal/MR-Gal** intracellularly with different pH. For fluorescence imaging, the excitation wavelength was 360-400 nm and 440 nm and emission channel 410-480 nm and 450-550 nm for Hoechst and **SP-Gal/SP-PEG**, respectively (scale bar: 100 mm, which is applicable to all images; the error bar represents s.d. (n = 3)).

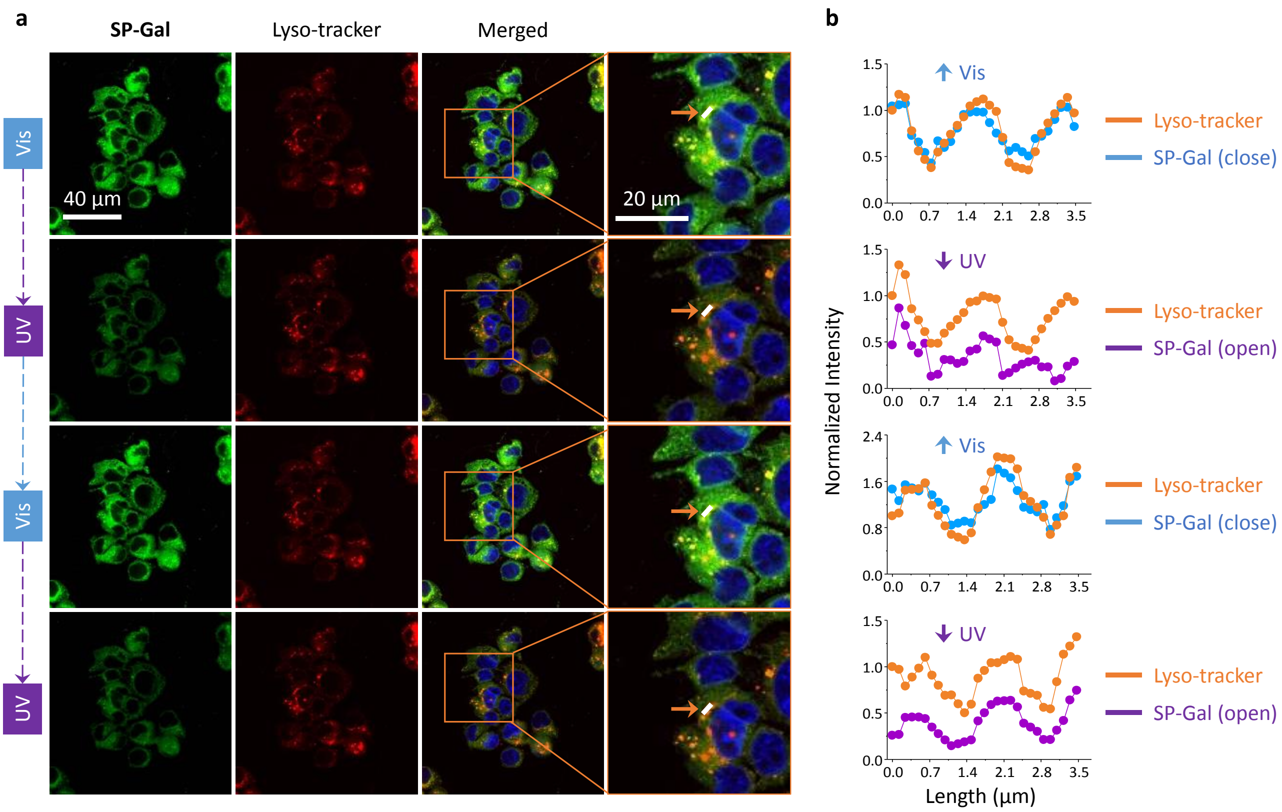

Supplementary Figure 13. UV/Vis cycling and colocalization of probe SP-Gal with lysosome probe Lyso-Tracker Red in Hep-G2 cells. (a) Fluorescence imaging of Hep-G2 cells with **SP-Gal** (40 µM, λ_{ex} = 440 nm, λ_{em} = 535 nm) and Lyso-Tracker Red (1 µM, λ_{ex} = 577 nm, λ_{em} = 590 nm). Scale bar represents 40 µm. (b) The "Focus" column represents an enlarged area of the "Merge" column as framed; the circles in the "Focus" column highlight the photochromic actions of **SP-Gal** in lysosomes.

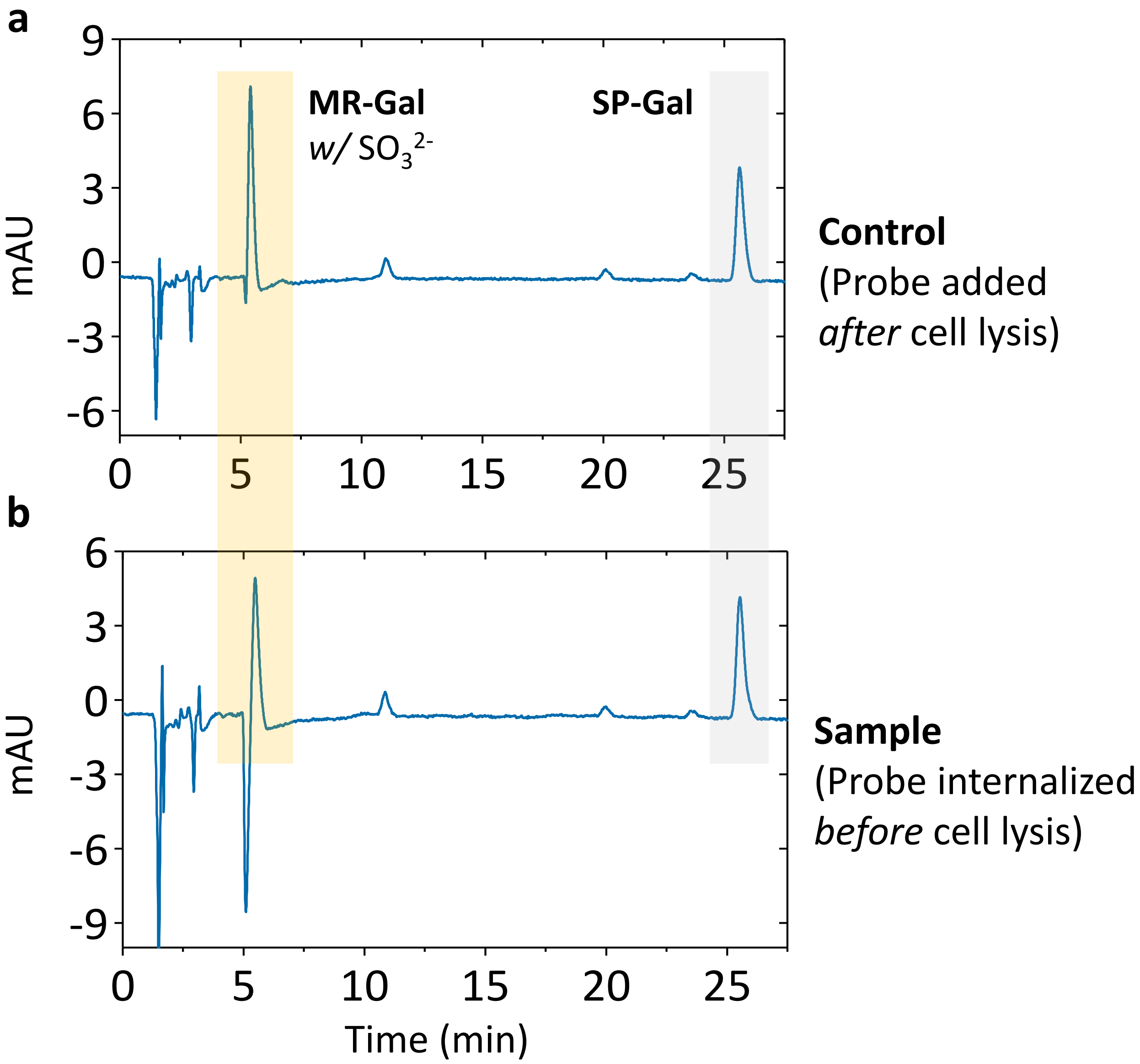

Supplementary Figure 14. High Performance Liquid Chromatography (HPLC) analysis of SP-Gal and MR-Gal-SO_3^{2-} in cell lysate. (a) **MR-Gal** (by treating **SP-Gal** with UV irradiation) was reacted with SO_3^{2-} in PBS, and then added to Hep-G2 cell lysate for HPLC analysis. (b) **SP-Gal** was internalized by live Hep-G2, irradiated by UV light (converting to **MR-Gal**), and then was added SO_3^{2-}. Subsequently, the cells were lysed for HPLC analysis. The Michael adduct of **MR-Gal** with (w/) SO_3^{2-} in **Sample** group corresponded with that in **Control** group. We note that the presence of **SP-Gal** trace might be a result of 1) an insufficient conversion of **SP-Gal** to **MR-Gal** and/or 2) a reversible conversion of the thermodynamically less stable **MR-Gal** to **SP-Gal** before reaction with sulfite.

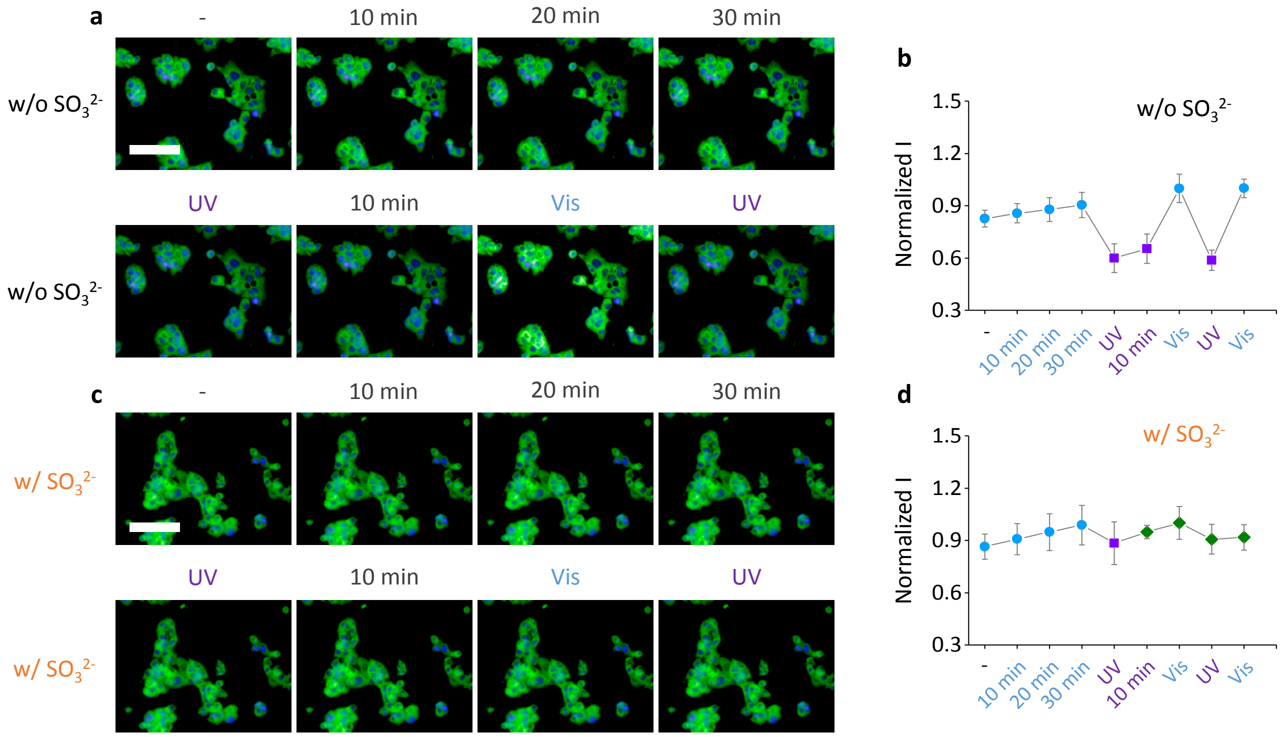

Supplementary Figure 15. UV/Vis cycling of SP-Gal (20 μM) in Hep-G2 (human hepatoma cell line). Fluorescence imaging (a) and quantification (b) of **SP-Gal** in Hep-G2 cells without (w/o) SO_3^{2-}. Fluorescence imaging (c) and quantification (d) of **SP-Gal** in Hep-G2 cells with (w/) SO_3^{2-} (80 μM). Scale bar: 100 mm, which is applicable to all images; the error bar represents s.d. (n = 3).

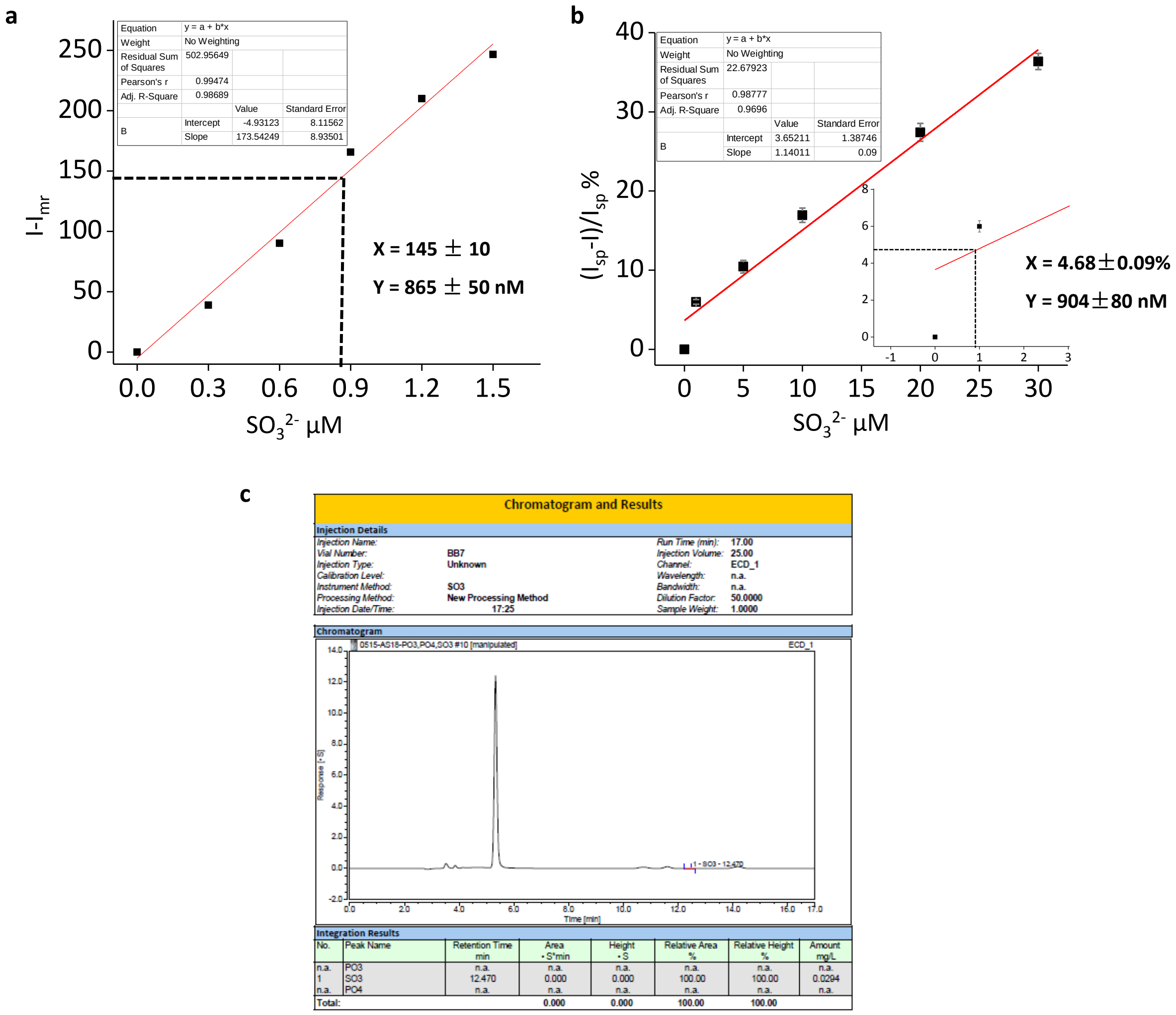

Supplementary Figure 16. (a) Quantification of lipopolysaccharide (LPS) induced endogenous sulfite in cell lysate by fluorescence calibration, where I_{mr} and I are the initial fluorescence intensity of **MR-Gal** and that of the probe after reaction with various concentrations of sulfite, respectively. (b) Quantification of lipopolysaccharide (LPS) induced endogenous sulfite in live Hep-G2 cells by fluorescence calibration, where I_{sp} and I are the initial fluorescence intensity of **SP-Gal** and that of **MR-Gal** (converted by UV) after reaction with various concentrations of sulfite, respectively. (c) Quantification of LPS induced endogenous sulfite in cell lysate by ion chromatography. Note that since the measurement was carried out under a 1:2 diluted lysate, the final result read from the chromatography measurement should be doubled. Also note that in the inset of (b), the intercept is around 3.70%, which might be a result of the complex intracellular environment that interferes with the sensing of analytes at very low concentrations. The error bar represents s.d. (n = 3).

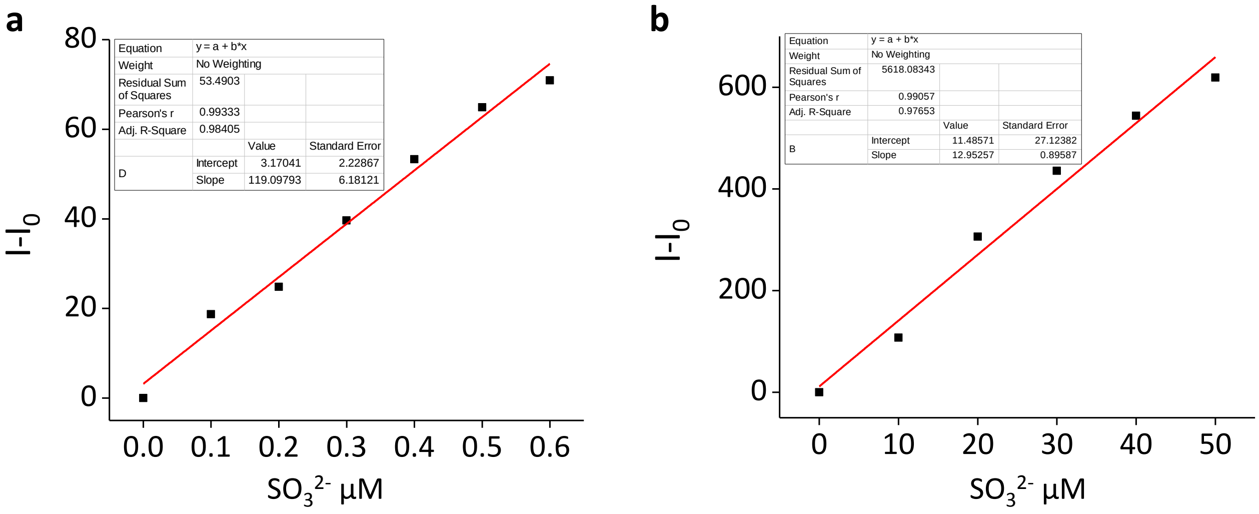

Supplementary Figure 17. (a) Plot for limit of detection (LOD) calculation of **SP-Gal** (0.1 μM in 0.05 M PBS, 1% DMSO, pH 7.4) with various concentrations of SO_3^{2-} (0.1-0.6 μM). (b) Plot for limit of detection (LOD) calculation of **SP-Gal** (10 μM in 0.05 M PBS, 1% DMSO, pH 7.4) with various concentrations of SO_3^{2-} (10-50 μM). The **SP-Gal** was first converted to **MR-Gal** under UV light before measuring. I_0 and I are the initial emission intensity of **MR-Gal** and that after treating with SO_3^{2-}, respectively.

For **SP-Gal** (0.1 μM in 0.05 M PBS, 1% DMSO, pH 7.4), a good linear relationship ($(I-I_0)$ versus SO_3^{2-} concentration, R^2 = 0.9841) was revealed in the detection range from 0 to 0.6 μM (Figure S17a). Under the present conditions (k = 3), the detection limit of **SP-Gal** was calculated as 2.6×10^{-8} M, using the formula $c_L = 3\sigma/k$.[1-2]

For **SP-Gal** (10 μM in 0.05 M PBS, 1% DMSO, pH 7.4), a good linear relationship ($(I-I_0)$ versus SO_3^{2-} concentration, R^2 = 0.9765) was revealed in the detection range from 0 to 50 μM (Figure S17b). Under the present conditions (k = 3), the detection limit of **SP-Gal** was calculated as 4.0×10^{-8} M, using the formula $c_L = 3\sigma/k$.

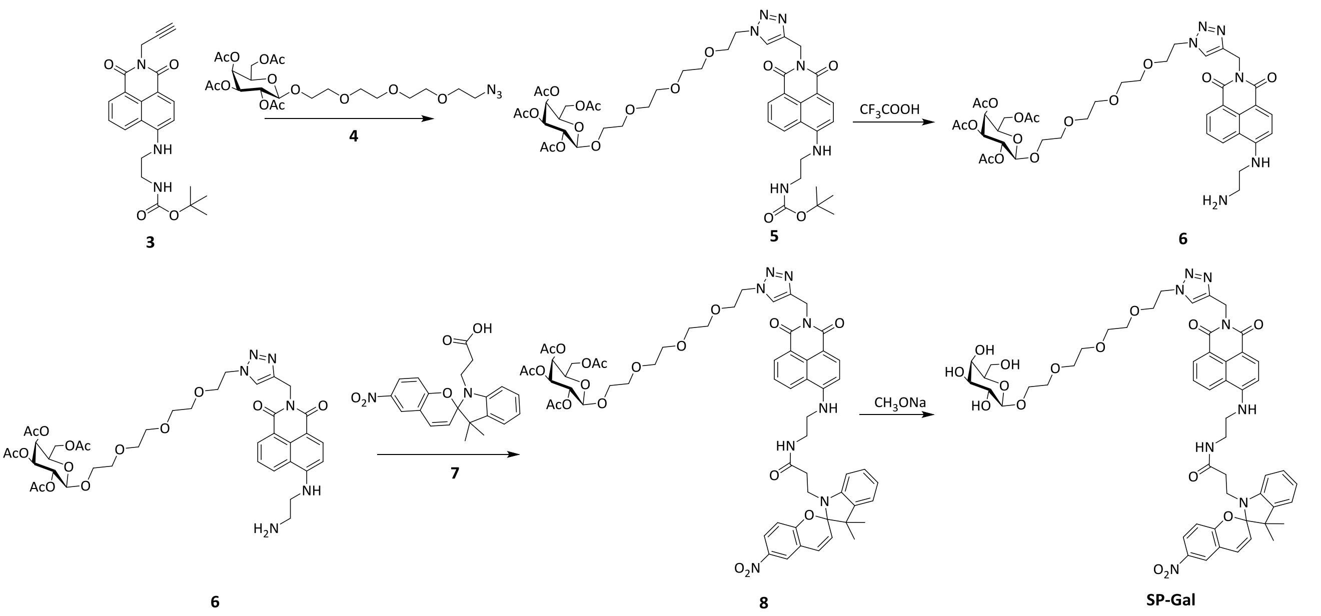

Supplementary Figure 18. Synthesis of **SP-Gal**.

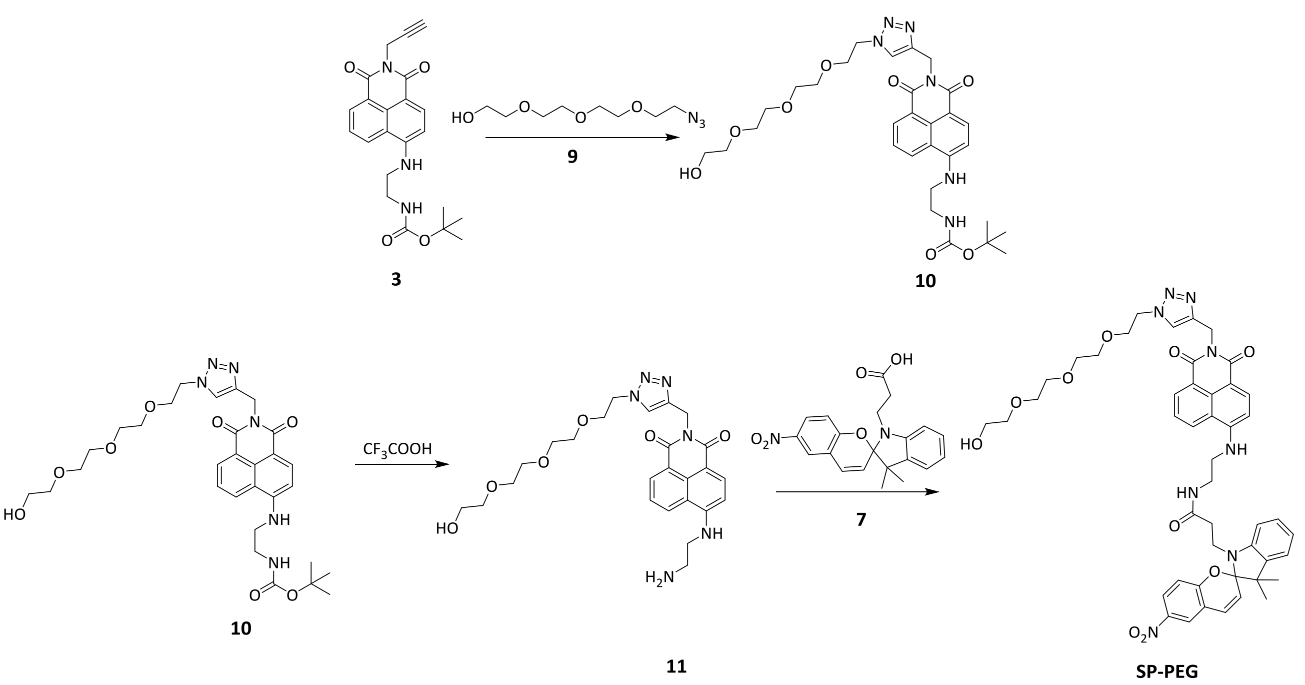

Supplementary Figure 19. Synthesis of **SP-PEG**.

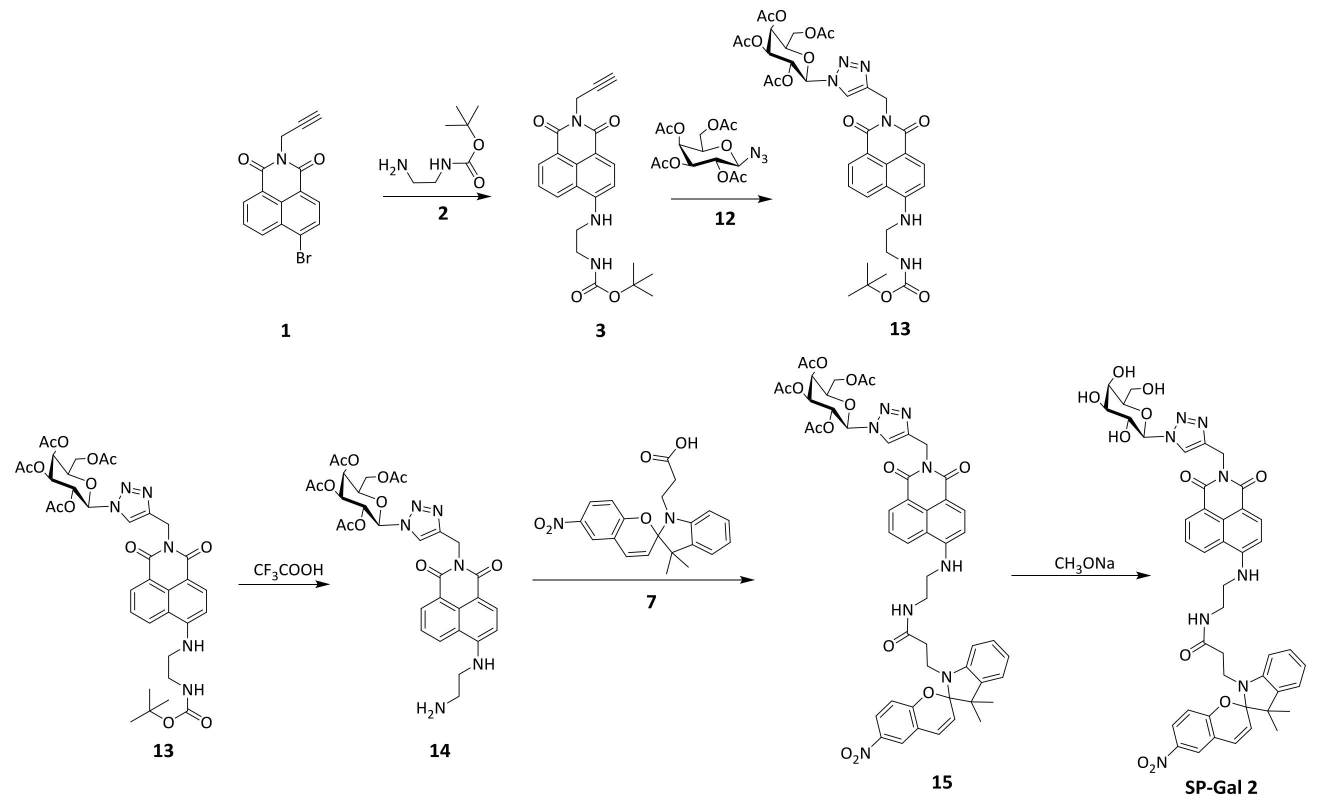

Supplementary Figure 20. Synthesis of **SP-Gal 2**.

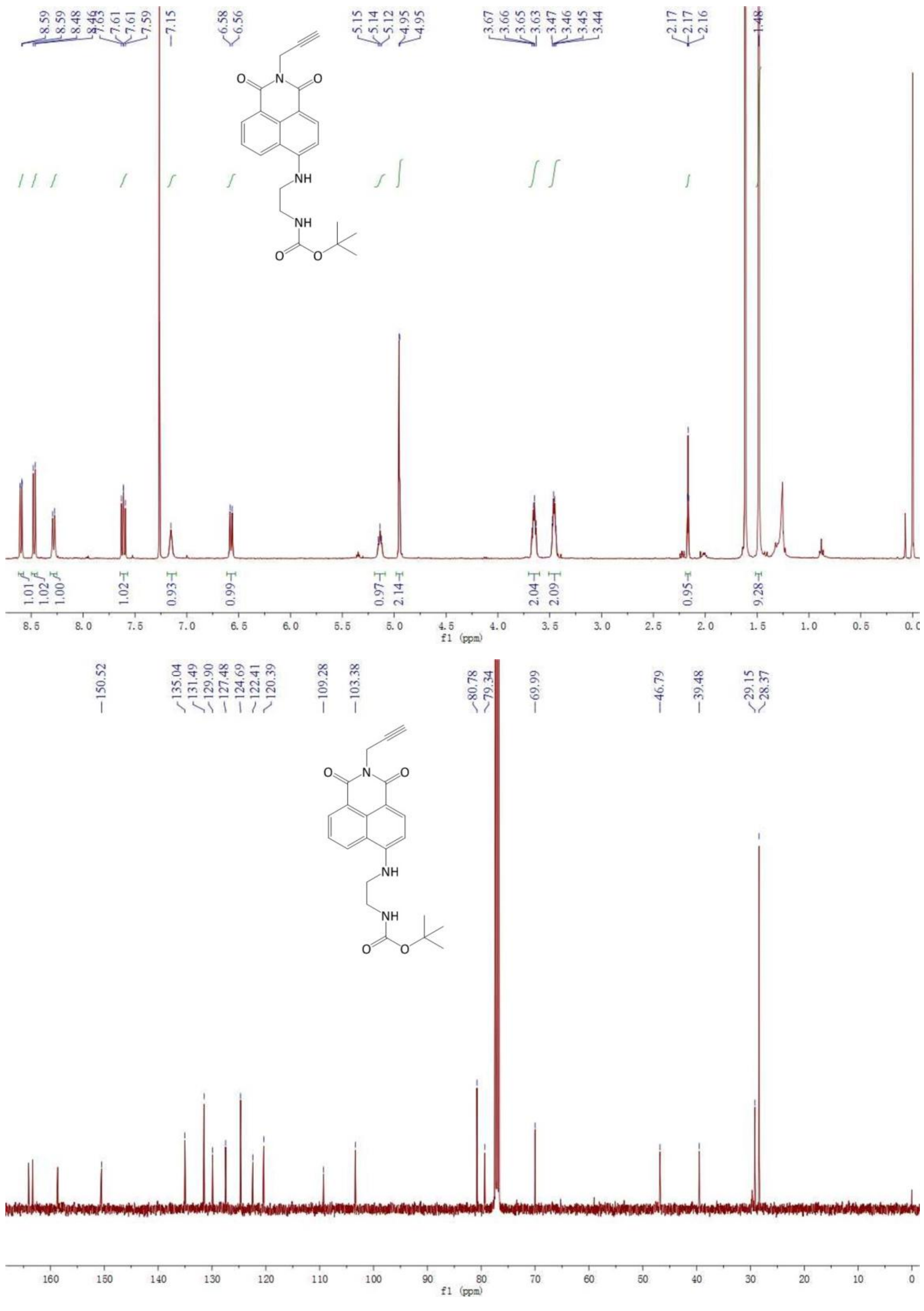

Supplementary Figure 21. Top: ^{1}H NMR of **3**. Bottom: ^{13}C NMR of **3**.

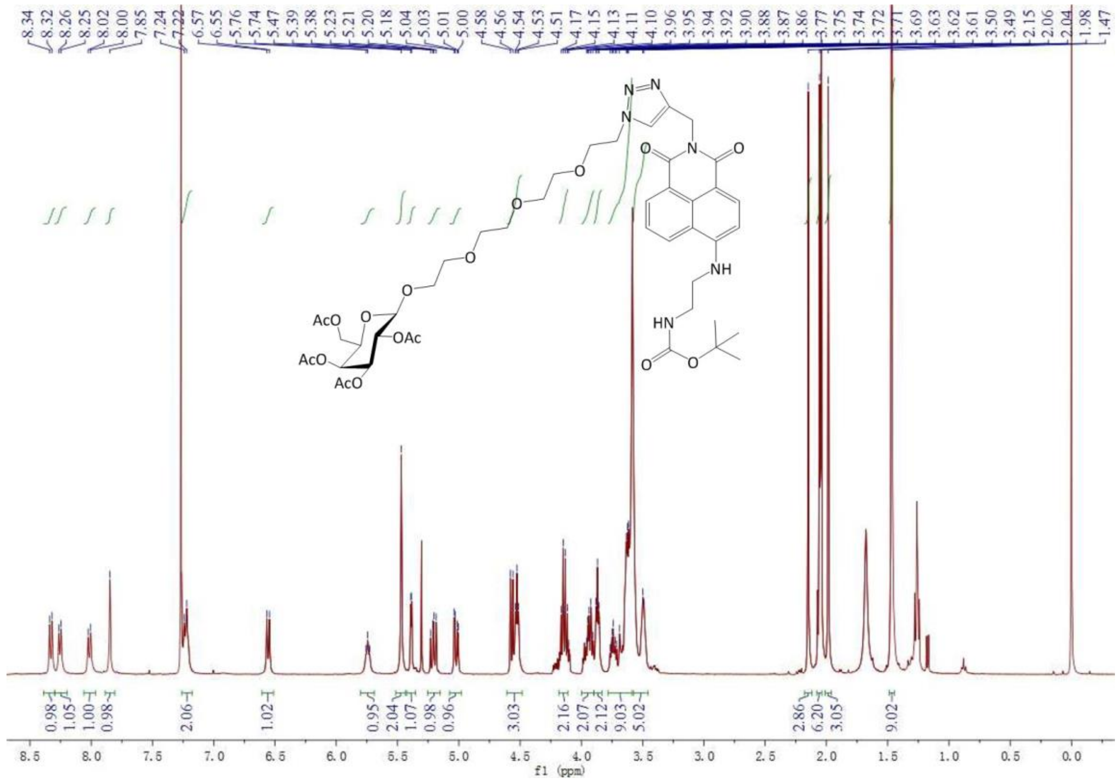

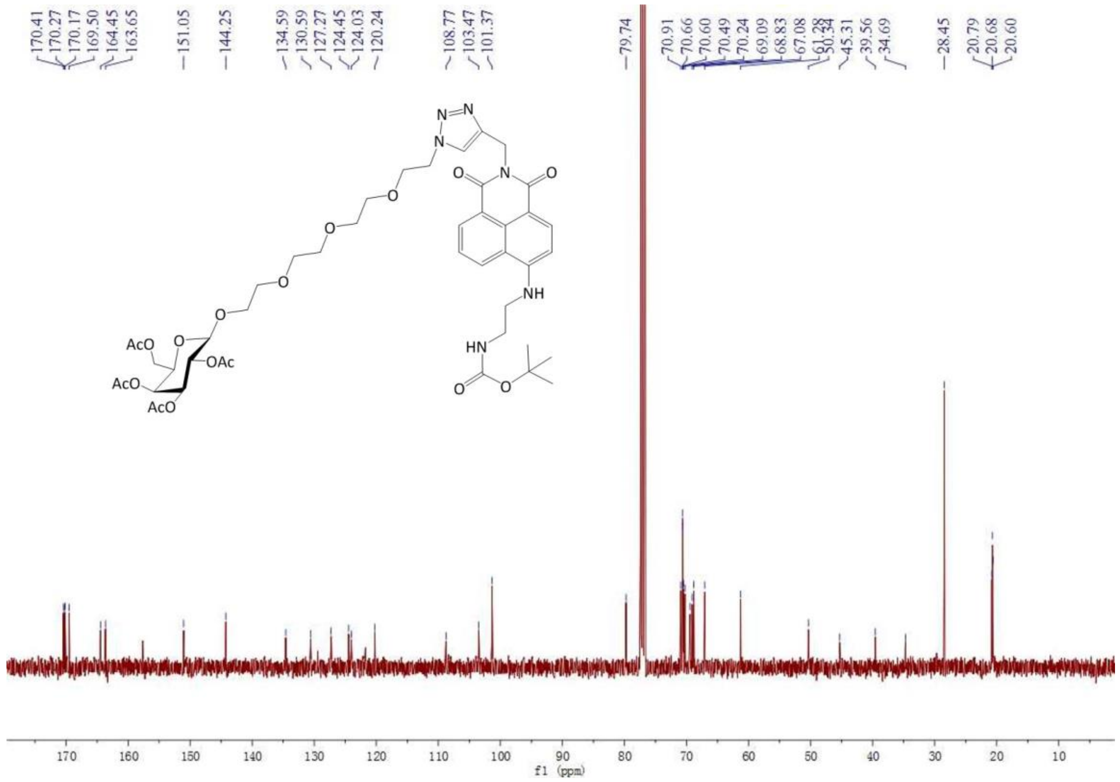

Supplementary Figure 22. Top: ^{1}H NMR of **5**. Bottom: ^{13}C NMR of **5**.

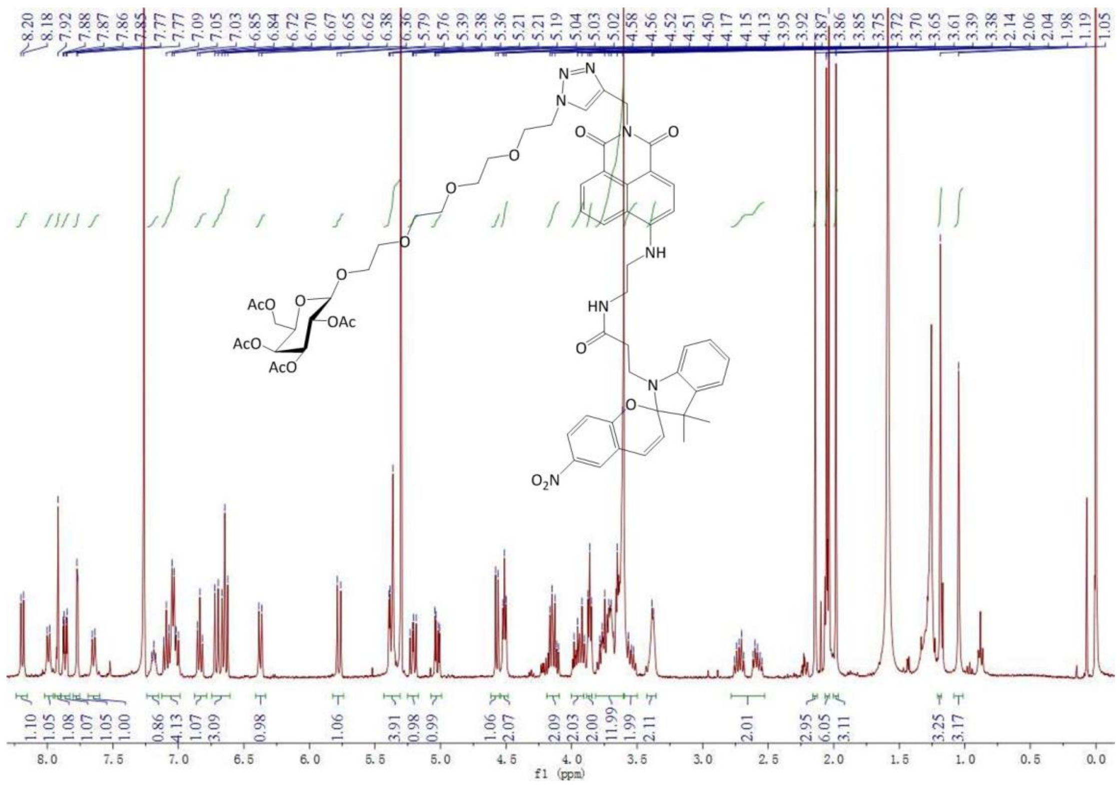

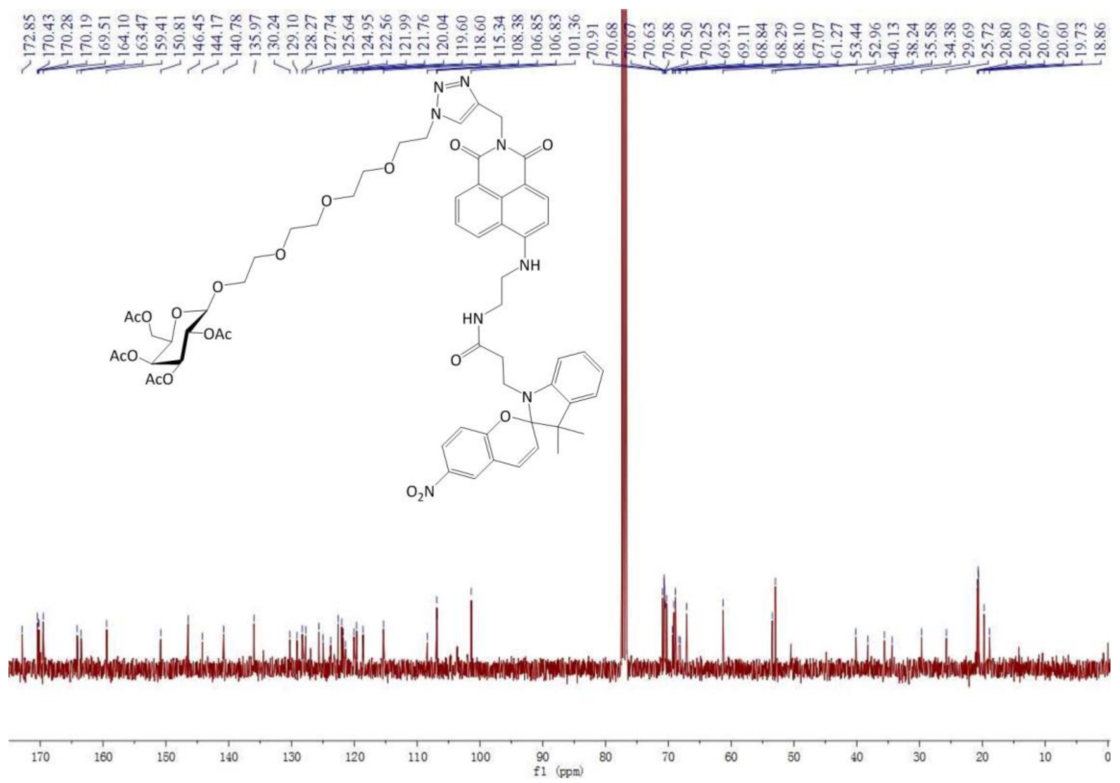

Supplementary Figure 23. Top: ^{1}H NMR of **8**. Bottom: ^{13}C NMR of **8**.

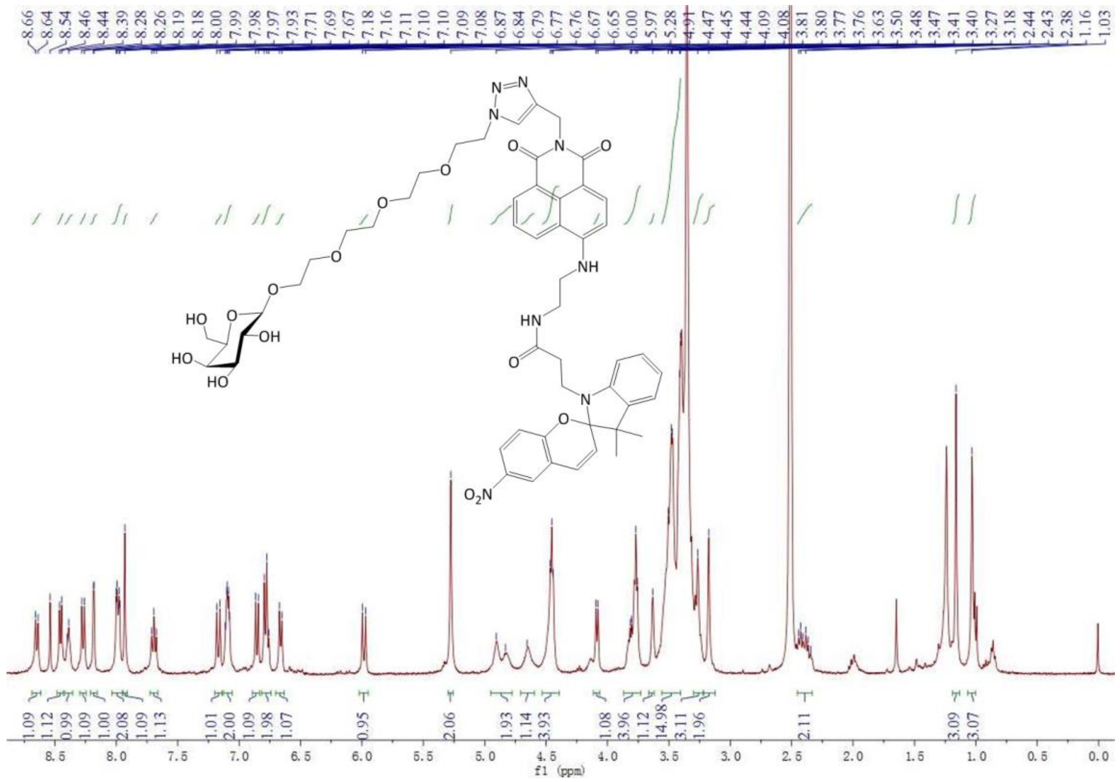

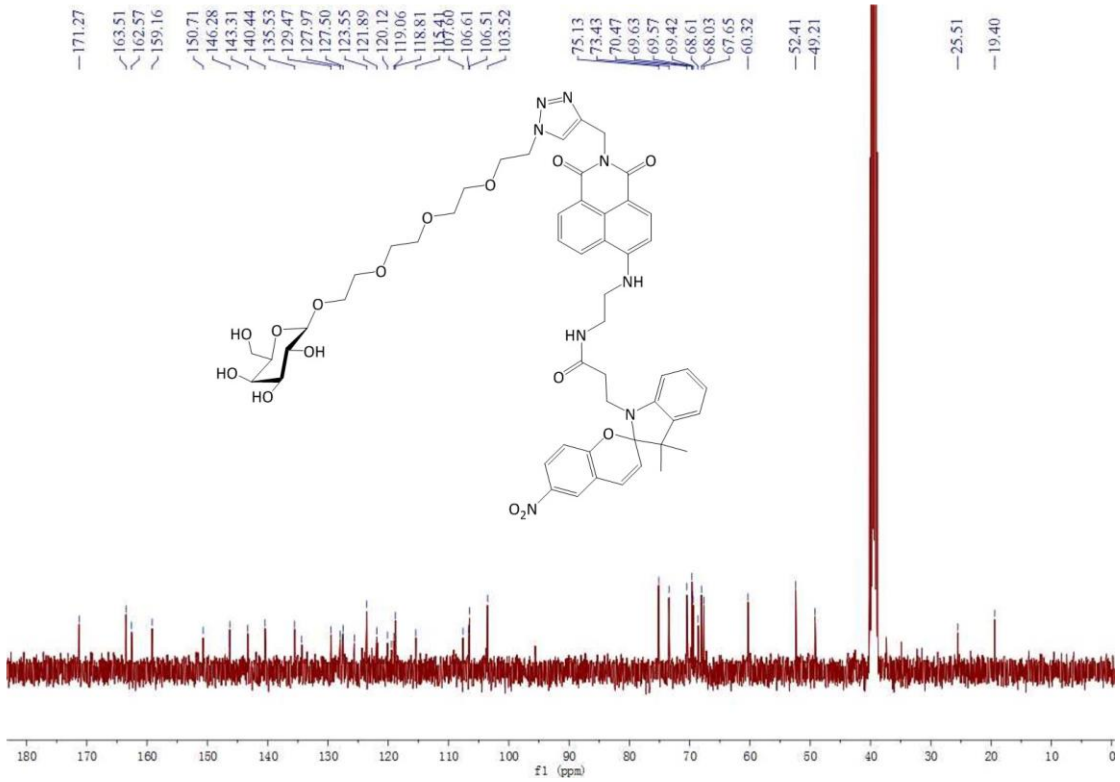

Supplementary Figure 24. Top: ^{1}H NMR of **SP-Gal**. Bottom: ^{13}C NMR of **SP- Gal**.

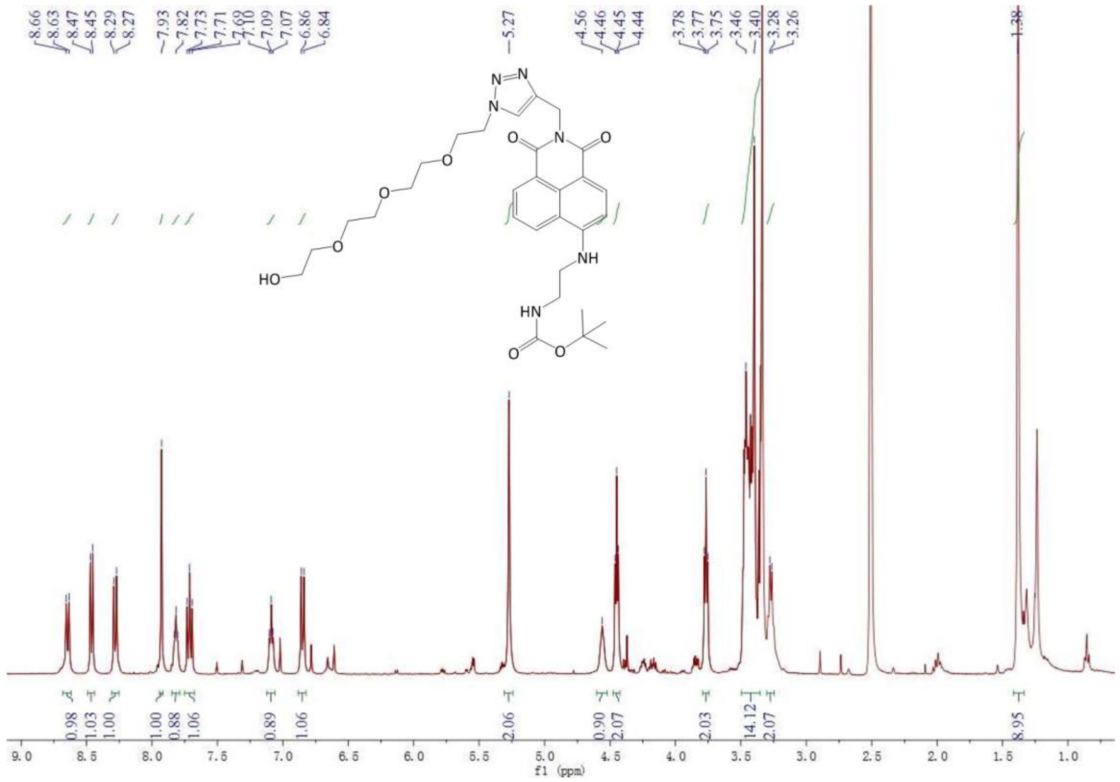

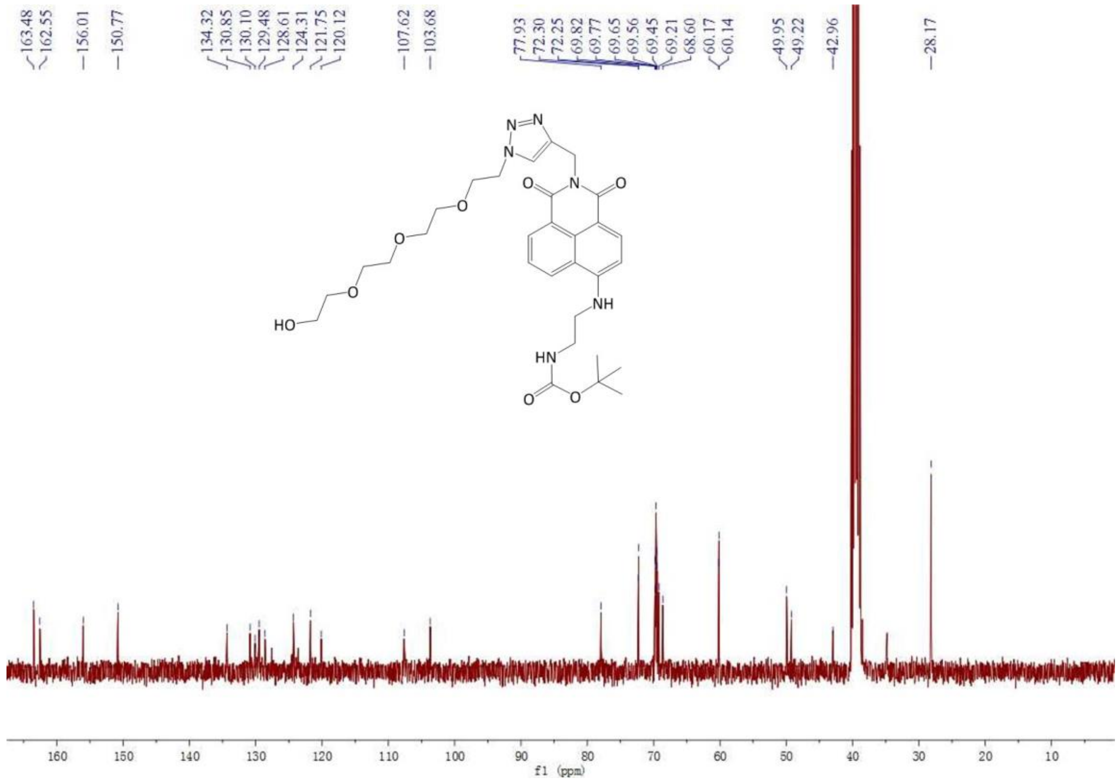

Supplementary Figure 25. Top: ^{1}H NMR of **10**. Bottom: ^{13}C NMR of **10**.

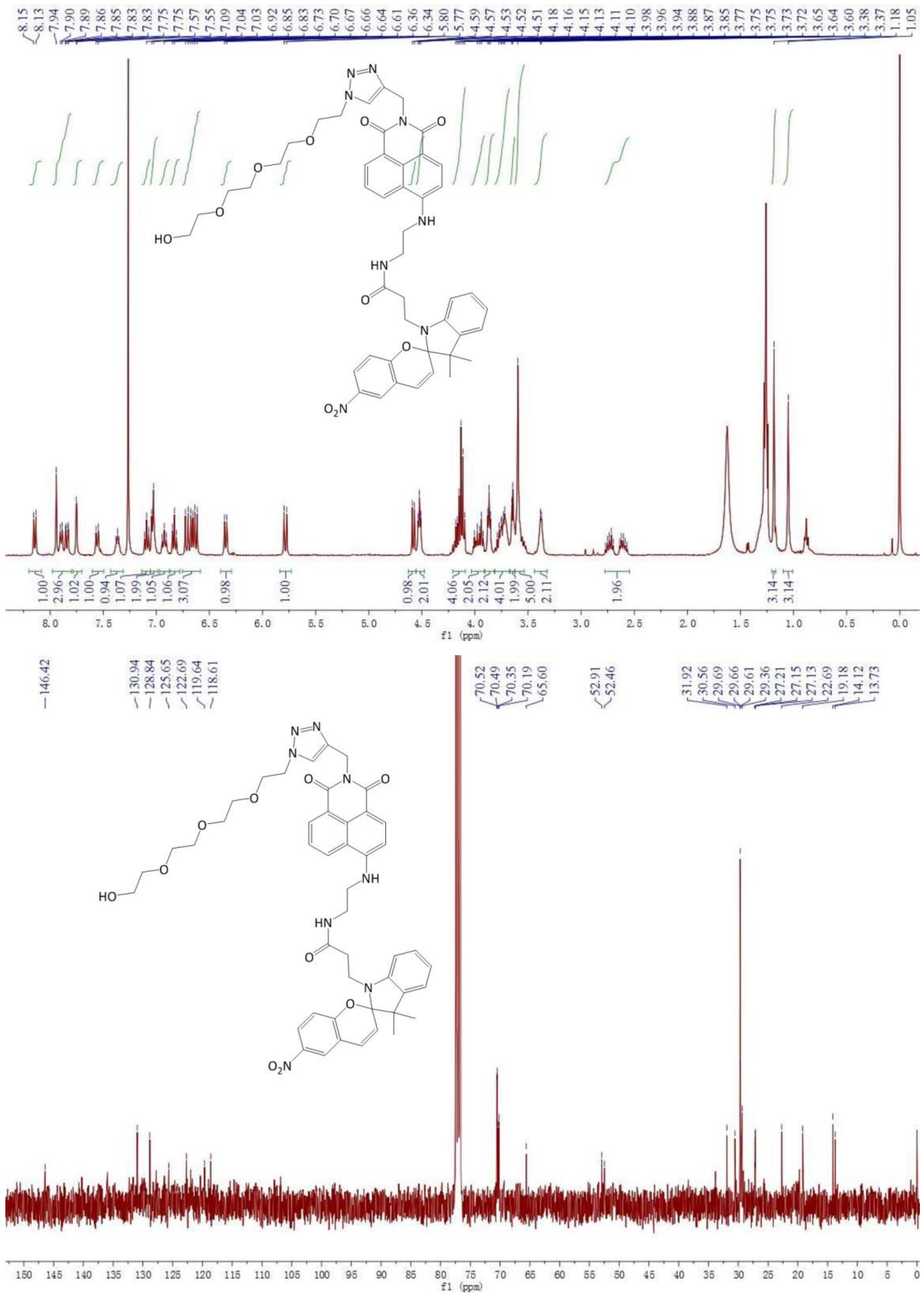

Supplementary Figure 26. Top: ^{1}H NMR of **SP-PEG**. Bottom: ^{13}C NMR of **SP-PEG**.

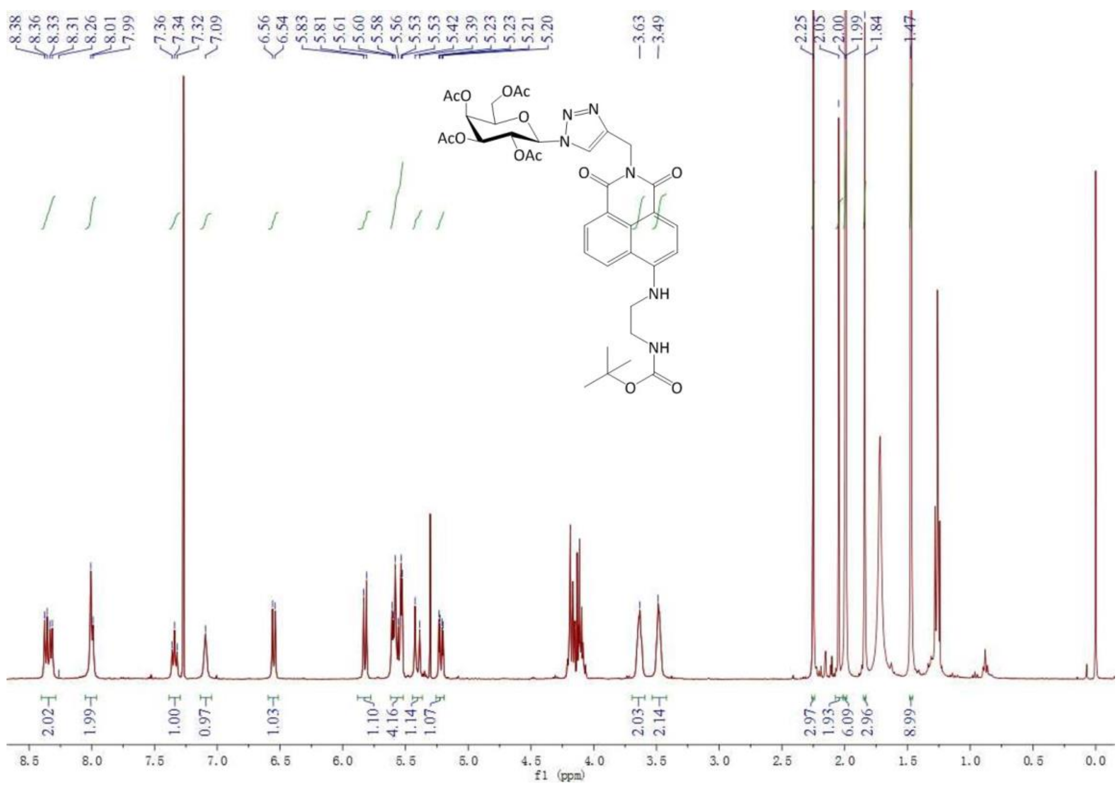

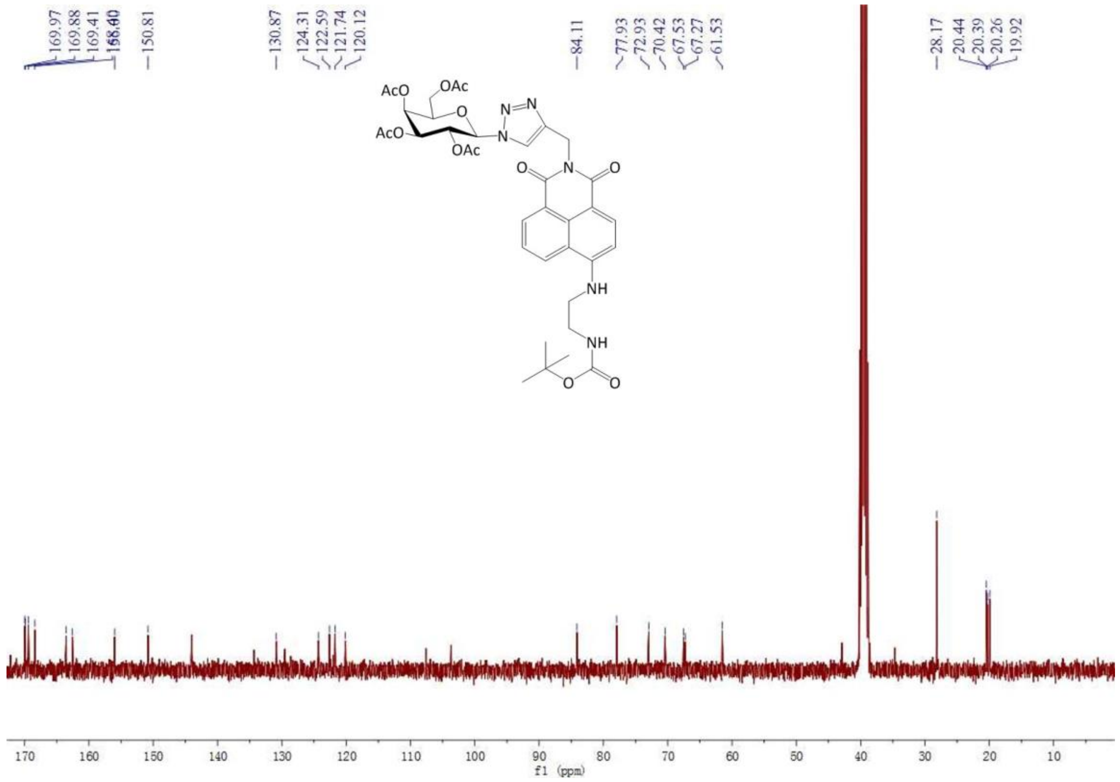

Supplementary Figure 27. Top: ^{1}H NMR of **13**. Bottom: ^{13}C NMR of **13**.

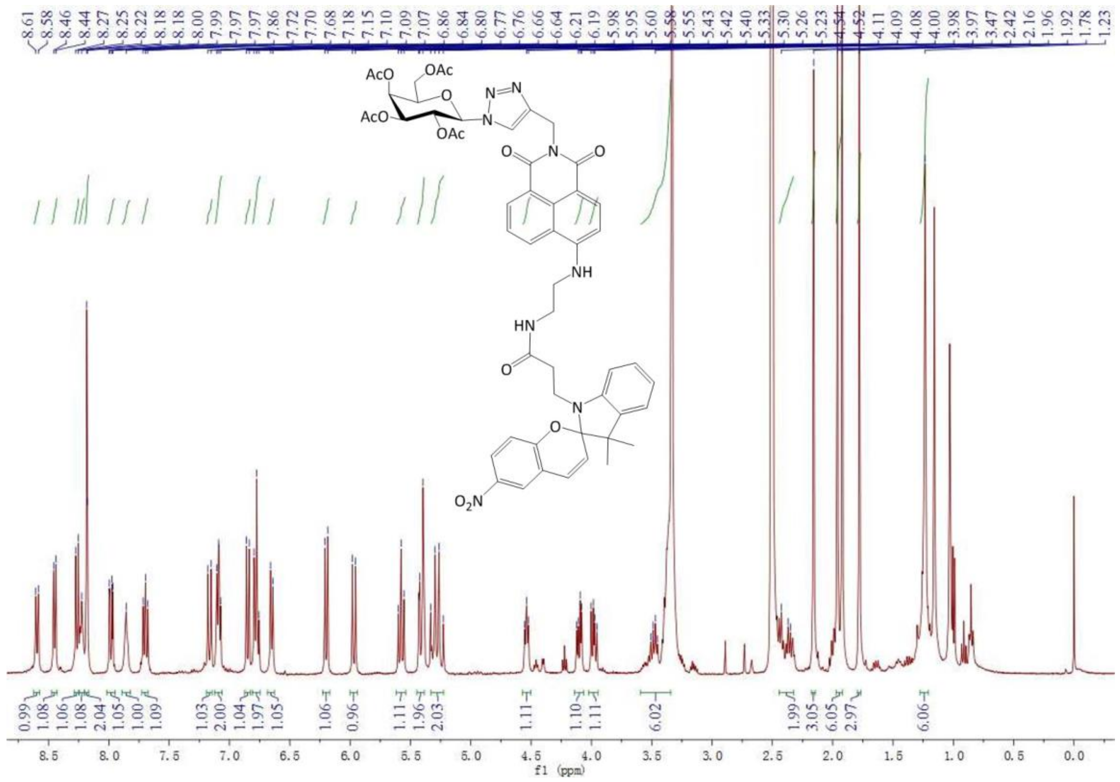

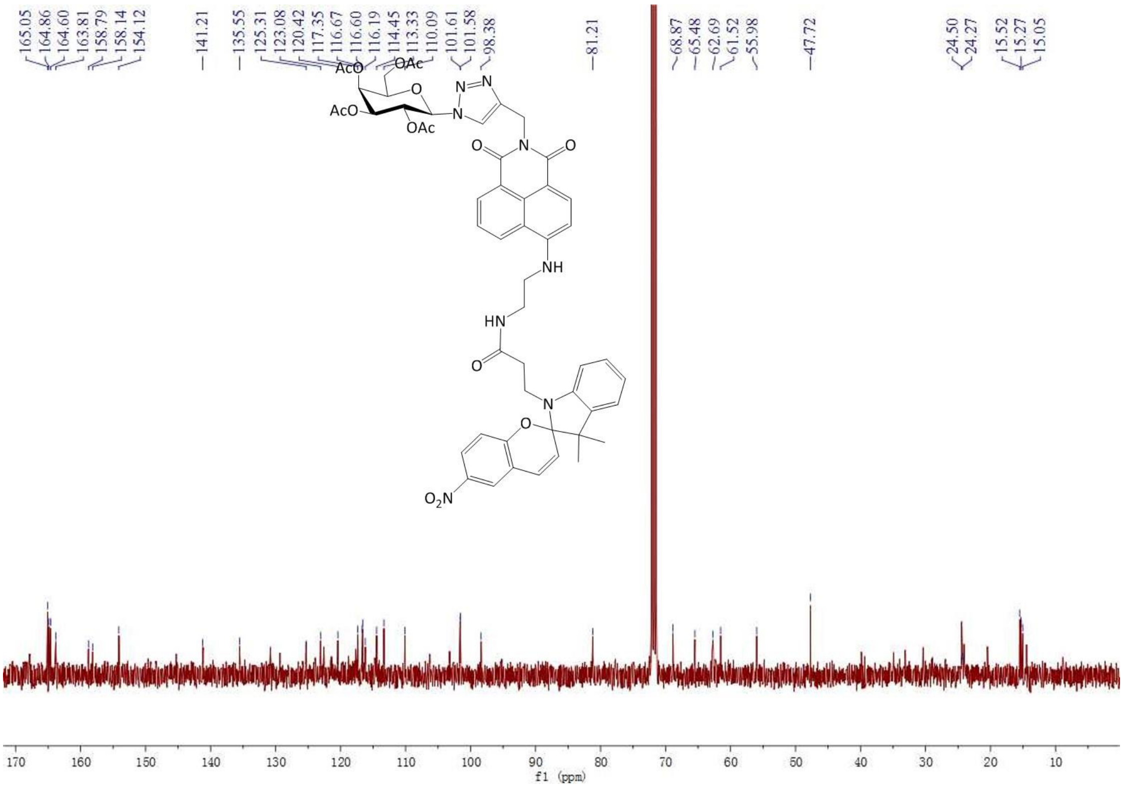

Supplementary Figure 28. Top: ^{1}H NMR of **15**. Bottom: ^{13}C NMR of **15**.

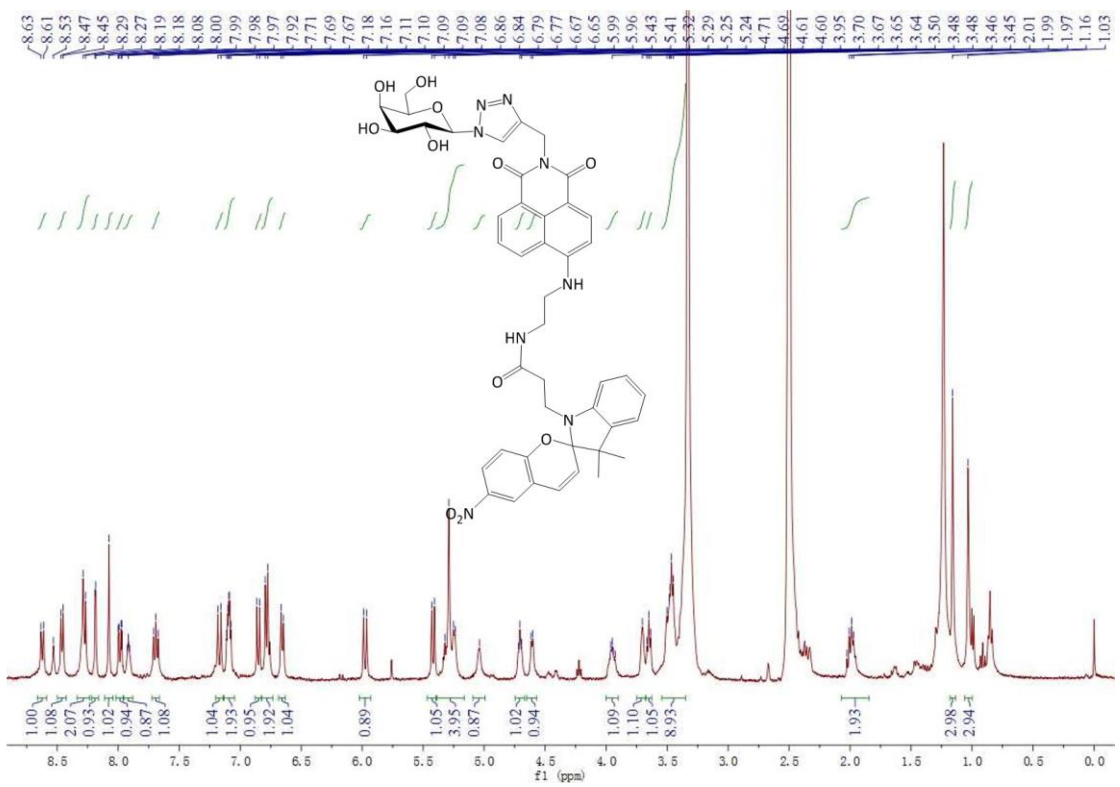

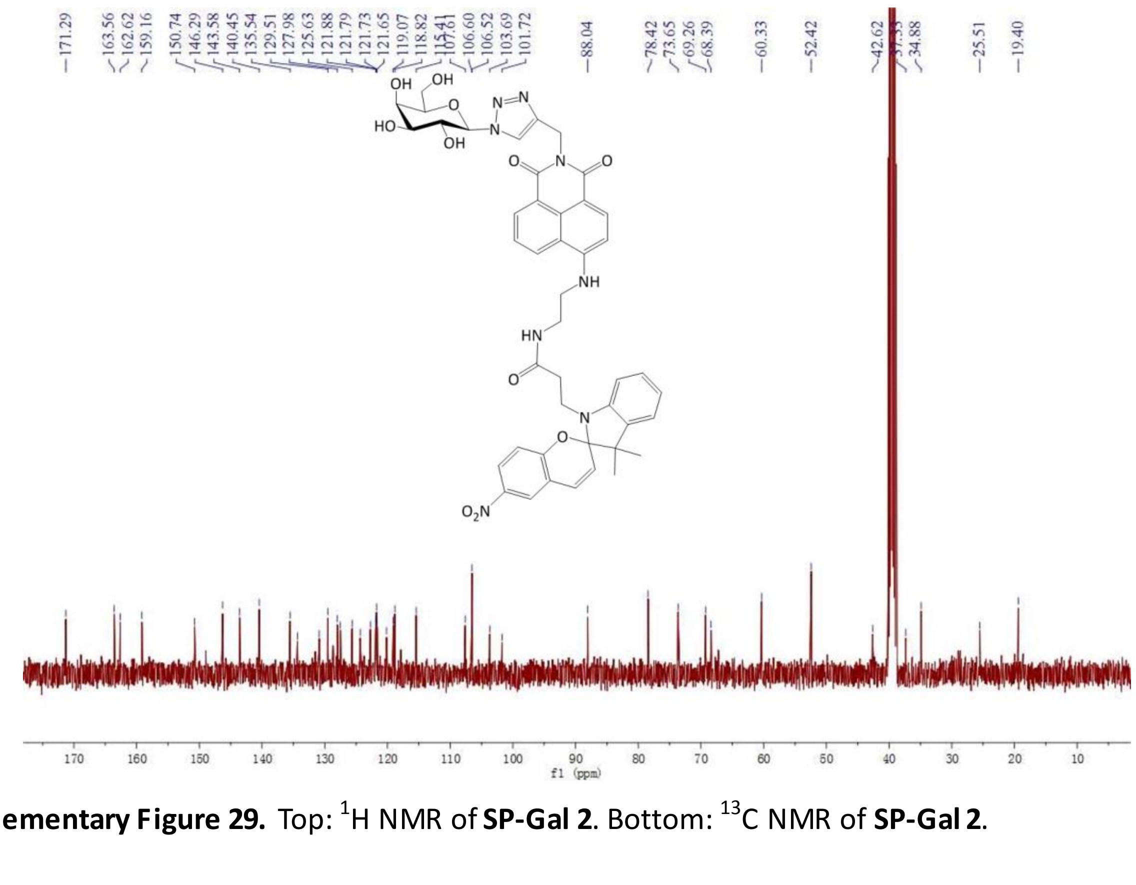

Supplementary Figure 29. Top: ^{1}H NMR of **SP-Gal 2**. Bottom: ^{13}C NMR of **SP-Gal 2**.

Elemental Composition Report

Single Mass Analysis

Tolerance = 50.0 PPM / DBE: min = -1.5, max = 100.0
Element prediction: Off
Number of isotope peaks used for i-FIT = 3

Monoisotopic Mass, Even Electron Ions
286 formula(e) evaluated with 1 results within limits (up to 1 closest results for each mass)
Elements Used:
C: 0-52 H: 0-100 N: 0-8 O: 0-15 Na: 0-1

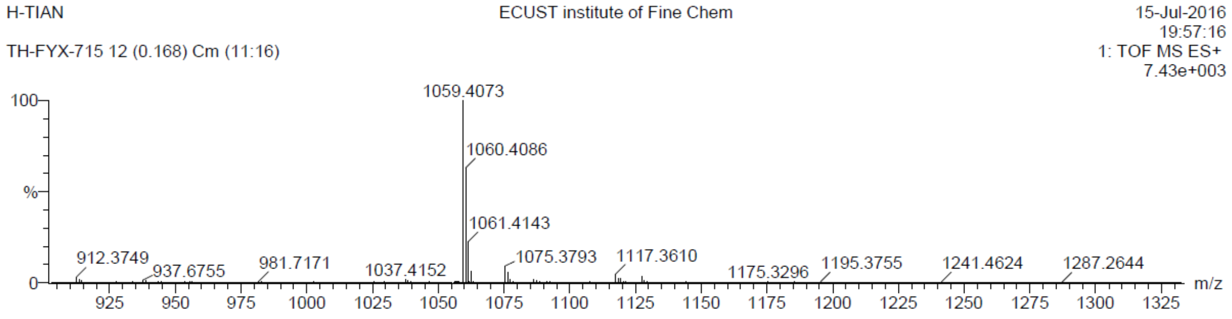

Minimum:				-1.5			
Maximum:		300.0	50.0	100.0			
Mass	Calc. Mass	mDa	PPM	DBE	i-FIT	i-FIT (Norm)	Formula
1059.4073	1059.4076	-0.3	-0.3	26.5	101.5	0.0	C52 H60 N8 O15 Na

Supplementary Figure 30. MS of **SP-Gal**.

Elemental Composition Report

Page 1

Single Mass Analysis

Tolerance = 500.0 PPM / DBE: min = -1.5, max = 100.0
Element prediction: Off
Number of isotope peaks used for i-FIT = 3

Monoisotopic Mass, Even Electron Ions
200 formula(e) evaluated with 47 results within limits (up to 1 closest results for each mass)
Elements Used:
C: 0-50 H: 0-50 N: 0-8 O: 0-11 Na: 0-1

H-TIAN ECUST institute of Fine Chem 03-Nov-2016 21:10:10

TH-FYX-004 34 (0.510) Cm (33:35) 1: TOF MS ES+ 8.05e+002

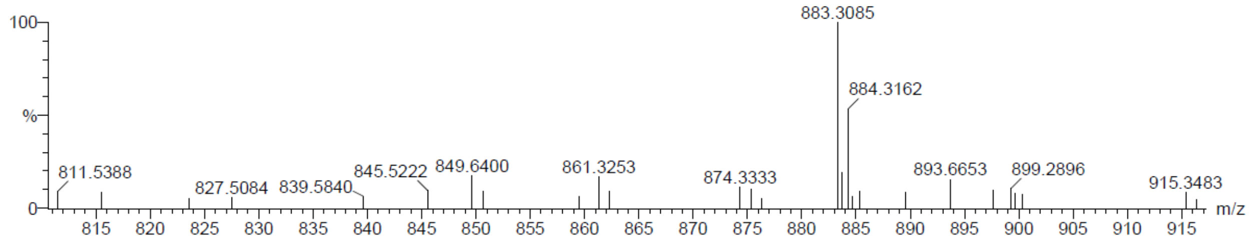

Minimum:				-1.5			
Maximum:		300.0	500.0	100.0			
Mass	Calc. Mass	mDa	PPM	DBE	i-FIT	i-FIT (Norm)	Formula
883.3085	883.3067	1.8	2.0	30.5	39.8	0.0	C49 H44 N6 O9 Na

Supplementary Figure 31. MS of **SP-Gal 2**.

Elemental Composition Report

Single Mass Analysis
Tolerance = 50.0 PPM / DBE: min = -1.5, max = 100.0
Element prediction: Off
Number of isotope peaks used for i-FIT = 3

Monoisotopic Mass, Even Electron Ions
208 formula(e) evaluated with 1 results within limits (up to 1 closest results for each mass)
Elements Used:
C: 0-46 H: 0-100 N: 0-8 O: 0-10 Na: 0-1

H-TIAN ECUST institute of Fine Chem 10-Oct-2016 21:36:00

TH-FYX-002 1 (0.118) Cm (1:4) 1: TOF MS ES+ 2.82e+002

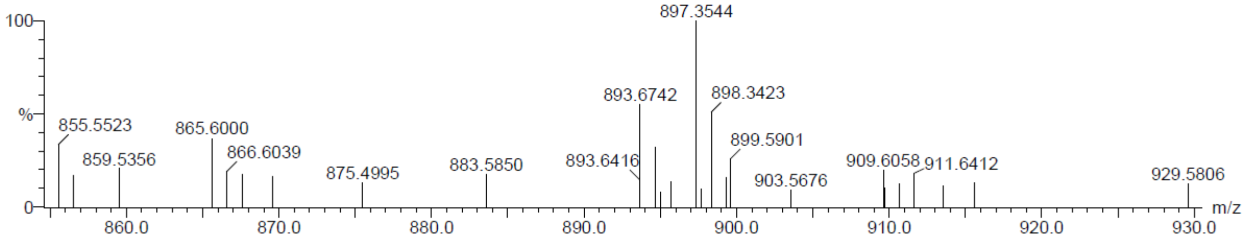

Minimum:				-1.5			
Maximum:		300.0	50.0	100.0			
Mass	Calc. Mass	mDa	PPM	DBE	i-FIT	i-FIT (Norm)	Formula
897.3544	897.3548	-0.4	-0.4	25.5	26.0	0.0	C46 H50 N8 O10 Na

Supplementary Figure 32. MS of **SP-PEG**.

Supplementary Table 1. Fluorescence quantum yield (Φ_F), photochromic quantum yield (Φ_P) and fluorescence lifetime (τ) of SP-Gal and MR-Gal.

	**SP-Gal**	**MR-Gal**
Φ_F (%)	32.10	7.81
Φ_P (%)	5.78	14.41
τ (ns)	4.14	1.41

Supplementary Note: Chemical Synthesis and Characterization.

*Synthesis of Compound **5**:* To a solution of compound **3** (0.20 g, 0.50 mmol) and compound **4** (0.33 g, 0.60 mmol), sodium ascorbate (0.40 g, 2.00 mmol) and $CuSO_4 \cdot 5H_2O$ (0.25 g, 1.00 mmol) were added, and the resulting mixture was stirred in DCM/H_2O (15:1, 10 mL) at room temperature for 12h. The resulting mixture was then diluted with dichloromethane and washed successively with water and brine. The combined organic layer was dried over $MgSO_4$, filtered and concentrated in vacuum. The resulting residue was purified by column chromatography on silica gel (dichloromethane/ethyl acetate = 6:1, v/v) to afford compound **5** (0.42 g, 90% yield). ^{1}H NMR (400 MHz, $CDCl_3$) δ 8.33 (d, *J* = 8.4 Hz, 1H), 8.25 (d, *J* = 7.2 Hz, 1H), 8.01 (d, *J* = 8.4 Hz, 1H), 7.85 (s, 1H), 7.23 (d, *J* = 8.0 Hz, 2H), 6.56 (d, *J* = 8.5 Hz, 1H), 5.74 (t, *J* = 5.7 Hz, 1H), 5.47 (s, 2H), 5.39 (d, *J* = 2.8 Hz, 1H), 5.21 (dd, *J* = 10.4, 8.0 Hz, 1H), 5.02 (dd, *J* = 10.5, 3.4 Hz, 1H), 4.61 – 4.48 (m, 3H), 4.14 (dd, *J* = 13.7, 6.9 Hz, 2H), 4.00 – 3.90 (m, 2H), 3.87 (t, *J* = 5.1 Hz, 2H), 3.78 – 3.59 (m, 9H), 3.49 (d, *J* = 4.1 Hz, 5H), 2.15 (s, 3H), 2.05 (d, *J* = 5.6 Hz, 6H), 1.98 (s, 3H), 1.47 (s, 9H). ^{13}C NMR (101 MHz, $CDCl_3$) δ 170.51, 169.98, 169.50, 164.45, 163.65, 151.05, 144.25, 134.59, 130.59, 127.27 124.92, 124.47, 124.24, 120.24, 108.77, 103.47, 101.37, 79.74, 70.91, 70.76, 70.43, 70.24, 69.42, 69.09, 68.83, 67.08, 61.28, 50.34, 45.31, 39.56, 34.69, 28.45, 20.89, 20.50. TOF MS ES$^+$ m/z: $[M + Na]^+$ calcd. for 965.3756, found 965.3756.

*Synthesis of Compound **8**:* Compound **5** (0.37 g, 0.40 mmol) was dissolved in TFA/DCM (1:4, 10 mL), and the resulting mixture was stirred at room temperature for 2 h. The solvent was evaporated to afford compound **6** (0.33 g), which was used directly for the next step without further purification.

To a solution of compound **7** (0.17 g, 0.47 mmol), were added 1-Ethyl-3-(3-dimethylaminopropyl)carbodiimide hydrochloride (EDCI, 0.26 g, 2 mmol) and 1-Hydroxybenzotriazole (HOBt, 0.38 g, 2 mmol), and the resulting mixture was stirred in dried DMF at 0 °C under Ar for 30 min. Then, compound **6** (0.33 g, 0.4 mmol) and triethylamine (0.30 mL) were sequentially added. The resulting mixture was stirred at room temperature for 24 h, and then poured into water and filtered to yield a yellowish powder. This powder was purified by column chromatography on silica (dichloromethane/methanol = 10:1 v/v) to afford compound **8** (0.24 g, 50% yield). ^{1}H NMR (400 MHz, $CDCl_3$) δ 8.19 (d, *J* = 8.4 Hz, 1H), 7.99 (d, *J* = 7.5 Hz, 1H), 7.92 (s, 1H), 7.86 (dd, *J* = 8.9, 2.7 Hz, 1H), 7.77 (d, *J* = 2.7 Hz, 1H), 7.65 (d, *J* = 8.4 Hz, 1H), 7.19 (t, *J* = 6.0 Hz, 1H), 7.13 – 6.99 (m, 4H), 6.84 (t, *J* = 7.4 Hz, 1H), 6.74 – 6.61 (m, 3H), 6.37 (d, *J* = 8.6 Hz, 1H), 5.77 (d, *J* = 10.3 Hz, 1H), 5.43 – 5.31 (m, 4H), 5.21 (dd, *J* = 10.4, 8.0 Hz, 1H), 5.02 (dd, *J* = 10.5, 3.4 Hz, 1H), 4.57 (d, *J* = 8.0 Hz, 1H), 4.51 (t, *J* = 5.0 Hz, 2H), 4.19 – 4.10 (m, 2H), 4.00 – 3.91 (m, 2H), 3.86 (t, *J* = 5.1 Hz, 2H), 3.82 – 3.60 (m, 12H), 3.60 – 3.50 (m, 2H), 3.38 (d, *J* = 4.3 Hz, 2H), 2.78 – 2.53 (m, 2H), 2.14 (s, 3H), 2.05 (d, *J* = 7.3 Hz, 6H), 1.98 (s, 3H), 1.19 (s, 3H), 1.05 (s, 3H). ^{13}C NMR (101 MHz, $CDCl_3$) δ

172.85, 170.53, 170.01, 164.10, 163.47, 159.41, 150.81, 146.45, 144.17, 140.78, 135.97, 130.24, 129.10, 128.27, 127.74, 125.64, 124.95, 123.74, 122.56, 121.99, 121.76, 121.37, 120.04, 119.60, 118.60, 115.34, 108.38, 106.84, 101.36, 70.91, 70.74, 70.31, 70.25, 69.32, 69.11, 68.84, 68.19, 67.07 (s), 61.27, 53.44, 52.96, 40.13, 38.24, 35.58, 34.38, 29.69, 25.72, 20.69, 19.73, 18.86. TOF MS ES$^+$ m/z: [M + Na]$^+$ calcd. for 1227.4498, found 1227.4514.

*Synthesis of Compound **SP-Gal**:* To a solution of compound **8** (0.15 g, 0.12 mmol) was added sodium methoxide (2.00 mg), and the resulting mixture was stirred in methanol/DCM (10:1, 8 mL) at room temperature for 2 h. Then, the solvent was evaporated and the resulting residue was purified by column chromatography on silica (dichloromethane-methanol = 3:1 v/v) to yield compound **SP-Gal** (0.11 g, 90% yield). ^{1}H NMR (400 MHz, DMSO-d_6) δ 8.65 (d, J = 8.4 Hz, 1H), 8.45 (d, J = 7.1 Hz, 1H), 8.39 (d, J = 4.8 Hz, 1H), 8.27 (d, J = 8.5 Hz, 1H), 8.18 (d, J = 2.6 Hz, 1H), 7.99 (dd, J = 8.8, 2.7 Hz, 2H), 7.93 (s, 1H), 7.69 (t, J = 7.9 Hz, 1H), 7.17 (d, J = 10.5 Hz, 1H), 7.13 – 7.06 (m, 2H), 6.85 (d, J = 9.0 Hz, 1H), 6.78 (t, J = 7.6 Hz, 2H), 6.66 (d, J = 7.9 Hz, 1H), 5.98 (d, J = 10.5 Hz, 1H), 5.28 (s, 2H), 4.87 (d, J = 30.1 Hz, 2H), 4.65 (brs, 1H), 4.53 – 4.39 (m, 4H), 4.09 (d, J = 6.9 Hz, 1H), 3.79 (dd, J = 18.5, 4.8 Hz, 4H), 3.63 (s, 1H), 3.47 (dd, J = 20.1, 16.9 Hz, 15H), 3.27 (s, 3H), 3.18 (s, 2H), 2.45 – 2.33 (m, 2H), 1.16 (s, 3H), 1.03 (s, 3H). ^{13}C NMR (101 MHz, DMSO-d_6) δ 171.27, 163.51, 162.57, 159.16, 150.71, 146.30, 143.31, 140.44, 135.53, 134.35, 129.47, 127.97, 127.52, 125.63, 123.55, 121.89, 120.12, 119.46, 119.06, 118.81, 115.41, 107.60, 106.56, 103.52, 75.13, 73.43, 70.47, 69.74, 69.32, 68.61, 68.03, 67.65, 60.32, 52.41, 49.21, 25.51, 19.40. TOF MS ES$^+$ m/z: [M + Na]$^+$ calcd. for 1059.4076, found 1059.4073.

*Synthesis of Compound **10**:* To a solution of compound **3** (0.20 g, 0.50 mmol) were added compound **9** (0.13 g, 0.60 mmol), sodium ascorbate (0.40 g, 2.00 mmol) and $CuSO_4 \cdot 5H_2O$ (0.25 g, 1.00 mmol), and the resulting mixture was stirred in DCM/H_2O (15:1, 10 mL) at room temperature for 12 h. The resulting mixture was then diluted with dichloromethane and washed successively with water and brine. The combined organic layer was dried over $MgSO_4$, filtered and concentrated in vacuum. The resulting residue was purified by column chromatography on silica gel (dichloromethane/ methanol = 6:1, v/v) to afford compound **10** (0.27 g, 90% yield). ^{1}H NMR (400 MHz, DMSO-d_6) δ 8.64 (d, J = 8.4 Hz, 1H), 8.46 (d, J = 7.2 Hz, 1H), 8.28 (d, J = 8.5 Hz, 1H), 7.93 (s, 1H), 7.82 (t, J = 4.9 Hz, 1H), 7.71 (t, J = 7.9 Hz, 1H), 7.09 (t, J = 5.6 Hz, 1H), 6.85 (d, J = 8.7 Hz, 1H), 5.27 (s, 2H), 4.56 (s, 1H), 4.45 (t, J = 5.2 Hz, 2H), 3.77 (t, J = 5.2 Hz, 2H), 3.43 (d, J = 25.2 Hz, 14H), 3.27 (d, J = 6.1 Hz, 2H), 1.38 (s, 9H). ^{13}C NMR (101 MHz, DMSO-d_6) δ 163.48, 162.55, 156.01, 150.77, 134.32, 130.85, 130.10, 129.48, 128.61, 124.31, 121.75, 120.12, 107.62, 103.68, 77.93, 72.28, 70.10, 69.27, 69.21, 68.60, 60.15, 49.95, 49.22, 42.96, 28.17. TOF MS ES+ m/z: [M + Na]+ calcd. for 635.2805, found 635.2799.

*Synthesis of Compound **SP-PEG**:* Compound **10** (0.20 g, 0.33 mmol) was dissolved in TFA/DCM

(1:4, 10 mL), and the resulting mixture was stirred at room temperature for 2 h. Then, solvent was evaporated to yield compound **11** (0.17 g), which was used directly for the next step without further purification.

To a solution of compound **7** (0.17 g, 0.47 mmol) were added 1-Ethyl-3-(3-dimethylaminopropyl)carbodiimide hydrochloride (EDCI, 0.26 g, 2 mmol) and 1-Hydroxybenzotriazole (HOBt, 0.38 g, 2 mmol), and the resulting mixture was stirred in dried DMF at 0°C under Ar for 30 min. Then compound **11** (0.17 g, 0.33 mmol) and triethylamine (0.30 mL) were sequentially added. The mixture was stirred at room temperature for 24 h, and then poured into water and filtered to yield a yellowish powder. This powder was purified by column chromatography on silica (dichloromethane/methanol = 10:1 v/v) to yield compound **SP-PEG** (0.10 g, 35% yield). ^{1}H NMR (400 MHz, $CDCl_3$) δ 8.14 (d, J = 8.4 Hz, 1H), 7.98 – 7.80 (m, 3H), 7.75 (d, J = 2.5 Hz, 1H), 7.56 (d, J = 8.5 Hz, 1H), 7.37 (t, J = 5.6 Hz, 1H), 7.09 (t, J = 7.4 Hz, 1H), 7.04 (d, J = 6.9 Hz, 2H), 6.93 (t, J = 7.8 Hz, 1H), 6.83 (t, J = 7.3 Hz, 1H), 6.75 – 6.58 (m, 3H), 6.35 (d, J = 8.6 Hz, 1H), 5.78 (d, J = 10.3 Hz, 1H), 4.58 (d, J = 7.9 Hz, 1H), 4.52 (t, J = 4.9 Hz, 2H), 4.21 – 4.09 (m, 4H), 4.03 – 3.91 (m, 2H), 3.87 (t, J = 5.0 Hz, 2H), 3.81 – 3.68 (m, 4H), 3.65 (d, J = 4.1 Hz, 2H), 3.60 (s, 5H), 3.38 (d, J = 4.0 Hz, 2H), 2.67 (ddt, J = 27.5, 14.0, 7.2 Hz, 2H), 1.18 (s, 3H), 1.05 (s, 3H). ^{13}C NMR (101 MHz, $CDCl_3$) δ 146.42, 130.94, 128.84, 125.65, 122.69, 119.64, 70.39, 65.60, 52.91, 52.46, 31.92, 30.56, 29.79, 29.53, 29.36, 27.32, 26.94, 22.69, 19.18, 14.12, 13.73. TOF MS ES+ m/z: [M + Na]+ calcd. for 897.3548, found 897.3544.

*Synthesis of Compound **3**:* To a solution of compound **1** (2.00 g, 6 mmol) was added compound **2** (4.50 g, 28 mmol), and the resulting mixture was stirred in 2-Methoxyethanol (45 mL) at 95 °C for 8 h. The mixture was then poured into water and filtered to yield a yellowish powder. This powder was purified by column chromatography on silica (dichloromethane) to yield compound **3** (1.20 g, 48% yield). ^{1}H NMR (400 MHz, $CDCl_3$) δ 8.60 (dd, J = 7.3, 0.9 Hz, 1H), 8.47 (d, J = 8.4 Hz, 1H), 8.28 (d, J = 8.4 Hz, 1H), 7.61 (dd, J = 8.3, 7.5 Hz, 1H), 7.15 (s, 1H), 6.57 (d, J = 8.4 Hz, 1H), 5.14 (t, J = 6.2 Hz, 1H), 4.95 (d, J = 2.4 Hz, 2H), 3.65 (dd, J = 10.3, 6.0 Hz, 2H), 3.46 (dd, J = 9.3, 4.2 Hz, 2H), 2.17 (t, J = 2.4 Hz, 1H), 1.48 (s, 9H). ^{13}C NMR (101 MHz, $CDCl_3$) δ 150.52, 135.04, 131.49, 129.90, 127.48, 124.69, 122.41, 120.39, 109.28, 103.38, 80.78, 79.34, 69.99, 46.79, 39.48, 29.15, 28.37. TOF MS ES^+ m/z: $[M + H]^+$ calcd. for 416.1586, found 416.1591.

*Synthesis of Compound **13**:* To a solution of compound **3** (0.20 g, 0.50 mmol) were added compound **12** (0.25 g, 0.60 mmol), sodium ascorbate (0.40 g, 2 mmol) and $CuSO_4{\cdot}5H_2O$ (0.25 g, 1 mmol), and the resulting mixture was stirred in DCM/H_2O (15:1, 10 mL) at room temperature for 12 h. The resulting mixture was then diluted with dichloromethane and washed successively with water and brine. The combined organic layer was dried over $MgSO_4$, filtered and concentrated in vacuum. The resulting residue was purified by column chromatography on silica gel

(dichloromethane/ethyl acetate = 5:1, v/v) to afford viscous compound **13** (0.35 g, 91% yield). ^{1}H NMR (400 MHz, $CDCl_3$) δ 8.34 (dd, J = 16.9, 7.8 Hz, 2H), 8.00 (d, J = 7.1 Hz, 2H), 7.34 (t, J = 7.8 Hz, 1H), 7.09 (s, 1H), 6.55 (d, J = 8.5 Hz, 1H), 5.82 (d, J = 9.3 Hz, 1H), 5.62 – 5.52 (m, 4H), 5.41 (d, J = 14.3 Hz, 1H), 5.22 (dd, J = 10.3, 3.3 Hz, 1H), 3.63 (s, 2H), 3.49 (s, 2H), 2.25 (s, 3H), 2.05 (s, 2H), 1.99 (d, J = 3.2 Hz, 6H), 1.84 (s, 3H), 1.47 (s, 9H). ^{13}C NMR (101 MHz, DMSO-d_6) δ 169.93, 169.41, 168.40, 163.53, 162.57, 156.00, 150.81, 130.87, 124.31, 122.59, 121.74, 120.12, 84.11, 77.93, 72.93, 70.42, 67.53, 67.27, 61.53, 28.17, 20.72, 20.16, 19.92. TOF MS ES$^+$ m/z: $[M + H]^+$ calcd. for 789.2708, found 789.2711.

*Synthesis of Compound **15**:* Compound **13** (0.30 g, 0.40 mmol) was dissolved in TFA/DCM (1:4, 10 mL) and stirred at room temperature for 2 h. Then, solvent was evaporated to yield compound **14** (0.26 g), which was used directly for the next step without further purification.

To a solution of compound **7** (0.17 g, 0.47 mmol) were added 1-Ethyl-3-(3-dimethylaminopropyl)carbodiimide hydrochloride (EDCI, 0.26 g, 2 mmol) and 1-Hydroxybenzotriazole (HOBt, 0.38 g, 2 mmol), and the resulting mixture was stirred in dried DMF at 0 °C under Ar for 30 min. Then, compound **14** (0.26 g, 0.4 mmol) and triethylamine (0.30 mL) were sequentially added. The mixture was stirred at room temperature for 24 h, and then poured into water and filtered to yield a yellowish powder. This powder was purified by column chromatography on silica (dichloromethane/methanol = 10:1 v/v) to yield compound **15** (0.20 g, 50% yield). ^{1}H NMR (400 MHz, DMSO-d_6) δ 8.59 (d, J = 8.5 Hz, 1H), 8.45 (d, J = 7.1 Hz, 1H), 8.26 (d, J = 8.5 Hz, 1H), 8.22 (s, 1H), 8.18 (d, J = 3.1 Hz, 2H), 7.98 (dd, J = 9.0, 2.8 Hz, 1H), 7.86 (s, 1H), 7.73 – 7.67 (m, 1H), 7.17 (d, J = 10.5 Hz, 1H), 7.12 – 7.06 (m, 2H), 6.85 (d, J = 9.0 Hz, 1H), 6.78 (t, J = 8.2 Hz, 2H), 6.65 (d, J = 7.9 Hz, 1H), 6.20 (d, J = 9.3 Hz, 1H), 5.97 (d, J = 10.4 Hz, 1H), 5.58 (t, J = 9.5 Hz, 1H), 5.45 – 5.39 (m, 2H), 5.33 – 5.23 (m, 2H), 4.57 – 4.50 (m, 1H), 4.10 (dd, J = 11.5, 4.9 Hz, 1H), 3.98 (dd, J = 11.5, 7.3 Hz, 1H), 3.48 (dd, J = 14.7, 7.3 Hz, 6H), 2.44 – 2.32 (m, 2H), 2.16 (s, 3H), 1.94 (d, J = 15.6 Hz, 6H), 1.78 (s, 3H), 1.23 (s, 6H). ^{13}C NMR (101 MHz, DMSO-d_6) δ 164.96, 164.60, 163.81, 158.79, 158.14, 154.12, 141.21, 135.55, 125.31, 123.08, 120.42, 117.35, 116.64, 116.19, 114.45, 113.33, 110.09, 101.60, 98.38, 81.21, 68.87, 65.48, 62.69, 61.52, 55.98, 47.72, 24.50, 24.27, 15.52, 15.16. TOF MS ES+ m/z: $[M + H]^+$ calcd. for 1051.3450, found 1051.3439.

*Synthesis of Compound **SP-Gal 2**:* To a solution of compound **15** (0.20 g) was added sodium methoxide (3.00 mg), and the resulting mixture was stirred in methanol/DCM (10:1, 8 mL) at room temperature for 2 h. The solvent was evaporated and the resulted residue was purified by column chromatography on silica (dichloromethane-methanol = 10:3 v/v) to yield **SP-Gal 2** (0.14 g, 84% yield). ^{1}H NMR (400 MHz, DMSO-d_6) δ 8.62 (d, J = 8.4 Hz, 1H), 8.46 (d, J = 7.3 Hz, 1H), 8.28 (d, J = 8.6 Hz, 2H), 8.19 (d, J = 2.7 Hz, 1H), 8.08 (s, 1H), 7.99 (dd, J = 8.9, 2.8 Hz, 1H), 7.96 – 7.88 (m, 1H), 7.72 – 7.66 (m, 1H), 7.17 (d, J = 10.5 Hz, 1H), 7.13 – 7.05 (m, 2H), 6.85 (d, J = 9.0 Hz, 1H), 6.78 (d, J = 8.1 Hz,

2H), 6.66 (d, J = 7.8 Hz, 1H), 5.98 (d, J = 10.4 Hz, 1H), 5.42 (d, J = 9.2 Hz, 1H), 5.40 – 5.16 (m, 4H), 5.04 (s, 1H), 4.71 (t, J = 5.2 Hz, 1H), 4.61 (d, J = 5.9 Hz, 1H), 3.94 (dd, J = 9.2, 6.0 Hz, 1H), 3.70 (s, 1H), 3.65 (t, J = 6.0 Hz, 1H), 3.54 – 3.35 (m, 9H), 2.00 (dd, J = 14.7, 7.0 Hz, 2H), 1.16 (s, 3H), 1.03 (s, 3H). ^{13}C NMR (101 MHz, DMSO-d_6) δ 171.29, 163.56, 162.62, 159.16, 150.74, 146.29, 143.58, 140.45, 135.54, 134.36, 130.88, 129.51, 127.98, 127.50, 125.63, 124.34, 122.72, 121.76, 120.14, 119.07, 118.82, 115.41, 107.61, 106.56, 103.69, 101.72, 88.04, 78.42, 73.65, 69.26, 68.39, 60.33, 52.42, 42.62, 37.33, 34.88, 25.51, 19.40. TOF MS ES$^+$ m/z: [M + H]$^+$ calcd. for 883.3067, found 883.3085.

Supplementary References:

1. Long, G. L. & Winefordner, J. D. Limit of detection. A closer look at the IUPAC definition. *Anal. Chem.* **55**, 712A-724A (1983).
2. Gilfrich, J. V. & Birks, L. S. Estimation of detection limits in X-ray fluorescence spectrometry. *Anal. Chem.* **56**, 77-79 (1984).
